# Neuroimaging and fluid biomarkers in Parkinson’s disease in an era of targeted interventions

**DOI:** 10.1038/s41467-024-49949-9

**Published:** 2024-07-05

**Authors:** Angeliki Zarkali, George E. C. Thomas, Henrik Zetterberg, Rimona S. Weil

**Affiliations:** 1 Dementia Research Centre, Institute of Neurology, UCL, London, UK; 2 Department of Psychiatry and Neurochemistry, Institute of Neuroscience and Physiology, the Sahlgrenska Academy at the University of Gothenburg, Mölndal, Sweden; 3 Clinical Neurochemistry Laboratory, Sahlgrenska University Hospital, Mölndal, Sweden; 4 Department of Neurodegenerative Disease, UCL Institute of Neurology, Queen Square, London, UK; 5 Hong Kong Center for Neurodegenerative Diseases, Clear Water Bay, Hong Kong, China; 6 Wisconsin Alzheimer’s Disease Research Center, University of Wisconsin School of Medicine and Public Health, University of Wisconsin-Madison, Madison, WI USA; 7 Department of Advanced Neuroimaging, UCL, London, UK; 8 Movement Disorders Centre, UCL, London, UK

**Keywords:** Parkinson's disease, Biomarkers, Parkinson's disease

## Abstract

A major challenge in Parkinson’s disease is the variability in symptoms and rates of progression, underpinned by heterogeneity of pathological processes. Biomarkers are urgently needed for accurate diagnosis, patient stratification, monitoring disease progression and precise treatment. These were previously lacking, but recently, novel imaging and fluid biomarkers have been developed. Here, we consider new imaging approaches showing sensitivity to brain tissue composition, and examine novel fluid biomarkers showing specificity for pathological processes, including seed amplification assays and extracellular vesicles. We reflect on these biomarkers in the context of new biological staging systems, and on emerging techniques currently in development.

## Abstract (web summary)

Biomarkers are needed in Parkinson’s for accurate diagnosis, to monitor progression and for targeted treatment. The authors reflect on recent imaging and fluid biomarkers for Parkinson’s and consider challenges in bringing them into clinical use.

## Introduction

Parkinson’s disease (PD) is the second most common neurodegenerative disorder, affecting over 6 million people worldwide^1^, with a marked increase in prevalence in the past 30 years^1^. It is characterised by rigidity, slowness and tremor, and causes disabling symptoms including dementia, depression and hallucinations. Although the pathological hallmark of PD is the accumulation of alpha-synuclein-containing Lewy bodies, most patients also have varying degrees of other pathological accumulations, including beta-amyloid plaques and tau-tangles, which are more strongly linked with dementia onset and poor outcomes^2^. A major challenge is the wide heterogeneity in initial presentation and rates of progression, which is partly underpinned by variation in underlying pathological accumulations^2^. This, coupled with a lack of sensitive and specific diagnostic markers, and quantitative biomarkers of progression, has hampered clinical trials for disease modification in PD^3^.

**Fig. 1:**
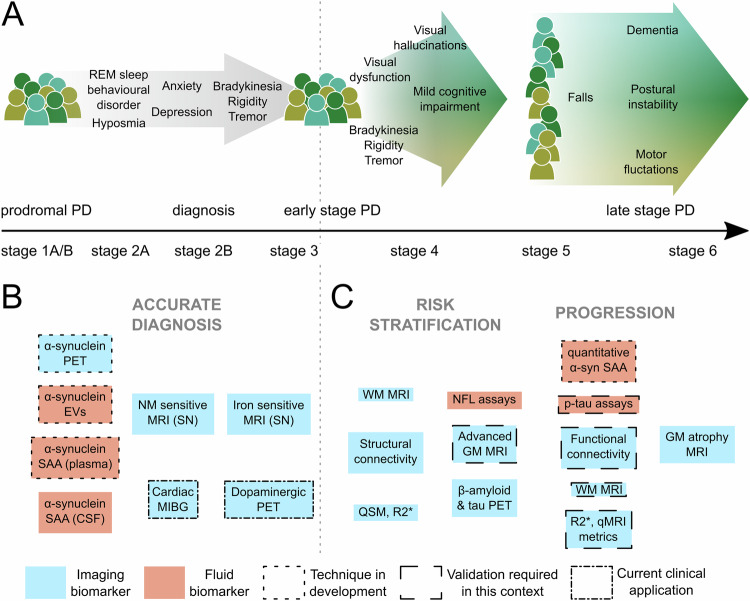
Fluid and imaging biomarkers over the course of Parkinson’s disease (PD). **A** Key clinical features from prodromal to late-stage PD, emphasising the high degree of heterogeneity. Below the black arrow are the biological stages of alpha-synuclein disease, as proposed by Simuni et al.^4^. Stages 1 A/B are characterised by biomarkers of neuronal alpha-synuclein and dopaminergic dysfunction, with clinical signs and symptoms emerging in stages 2 A/B and functional impairment emerging from stage 3. **B**, **C** Key fluid and imaging biomarkers in PD, in orange and blue boxes, respectively. **B** Fluid and imaging biomarkers for accurate diagnosis. Techniques to the left have increased sensitivity at earlier as well as later stages. **C** Fluid and imaging biomarkers for risk stratification and tracking disease progression in PD. Techniques to the left have greater utility in risk stratification for poor outcomes or faster progression. Techniques to the right show utility or potential for tracking disease progression. Dotted borders indicate techniques still in development. Dashed borders indicate techniques that may be well-established in other contexts but require further validation for these uses. α-syn alpha-synuclein; CSF cerebrospinal fluid; EVs Extracellular vesicles; GM grey matter; MIBG meta-iodoenzylguanidine scintigraphy; NFL neurofilament light chain; NM neuromelanin; PET positron emission tomography; qMRI quantitative MRI; QSM quantitative susceptibility mapping; REM rapid eye movement; SAA seed amplification assay; SN substantia nigra.; WM white matter.

Quantitative biomarkers are important in PD for three key reasons: i) for early and accurate diagnostic certainty in the clinic and for inclusion in clinical trials; ii) to monitor disease progression and predict outcomes; and iii) to stratify patients with differing underlying pathologies to be targeted for distinct treatments (see Fig. 1). They can also improve trial efficiency by showing evidence of target engagement as well as aid our understanding of underlying pathological mechanisms of PD spurring further treatment into potential therapeutic targets. The need for disease-specific biomarkers has been heightened further by calls for biological staging systems in PD^4,5^.

Until recently, neuroimaging measures have been limited as markers for diagnosis and progression in PD. DAT SPECT imaging to detect integrity of striatal dopaminergic terminals can support diagnosis but shows minimal progression over time^6^. MRI was previously unhelpful in diagnosis and monitoring of PD, with conventional measures such as brain atrophy showing inconsistent patterns in PD, and often appearing normal until late stages^7^. Fluid measures of alpha-synuclein levels have, until recently, shown inconsistent relationships with Lewy body pathology and disease presence or progression^8,9^. Instead, novel imaging markers sensitive to brain tissue composition^10^ are emerging, showing stronger relationships with disease severity. Novel measures of alpha-synuclein including its aggregation^11^, and CSF and plasma measures of other proteins^12–14^ also show promise as diagnostic and progression markers in PD and in related conditions such as dementia with Lewy bodies (DLB)^15^.

New treatments for neurodegenerative diseases are emerging, with some showing potential application in PD. These include anti-amyloid therapies^16,17^, and the p38 alpha-kinase inhibitor, neflamapimod^18^. Such interventions will require robust biomarkers to stratify patients for inclusion and as outcome measures to track responses to treatment. Here we provide a perspective of new and emerging neuroimaging and fluid biomarkers in PD. We consider their application for early and accurate diagnosis, in the context of recent proposals of staging systems for PD; in monitoring disease progression in PD; in stratifying patients for more precise treatment; and into quantifying target engagement, both in the clinic and in research. Finally, we consider novel approaches such as high-field MRI and multimodal information from wider body systems that show promise for identifying high-risk groups for more targeted therapeutic interventions.

## Biomarkers for early and accurate diagnosis

### Imaging biomarkers

#### Dopaminergic imaging

**Fig. 2:**
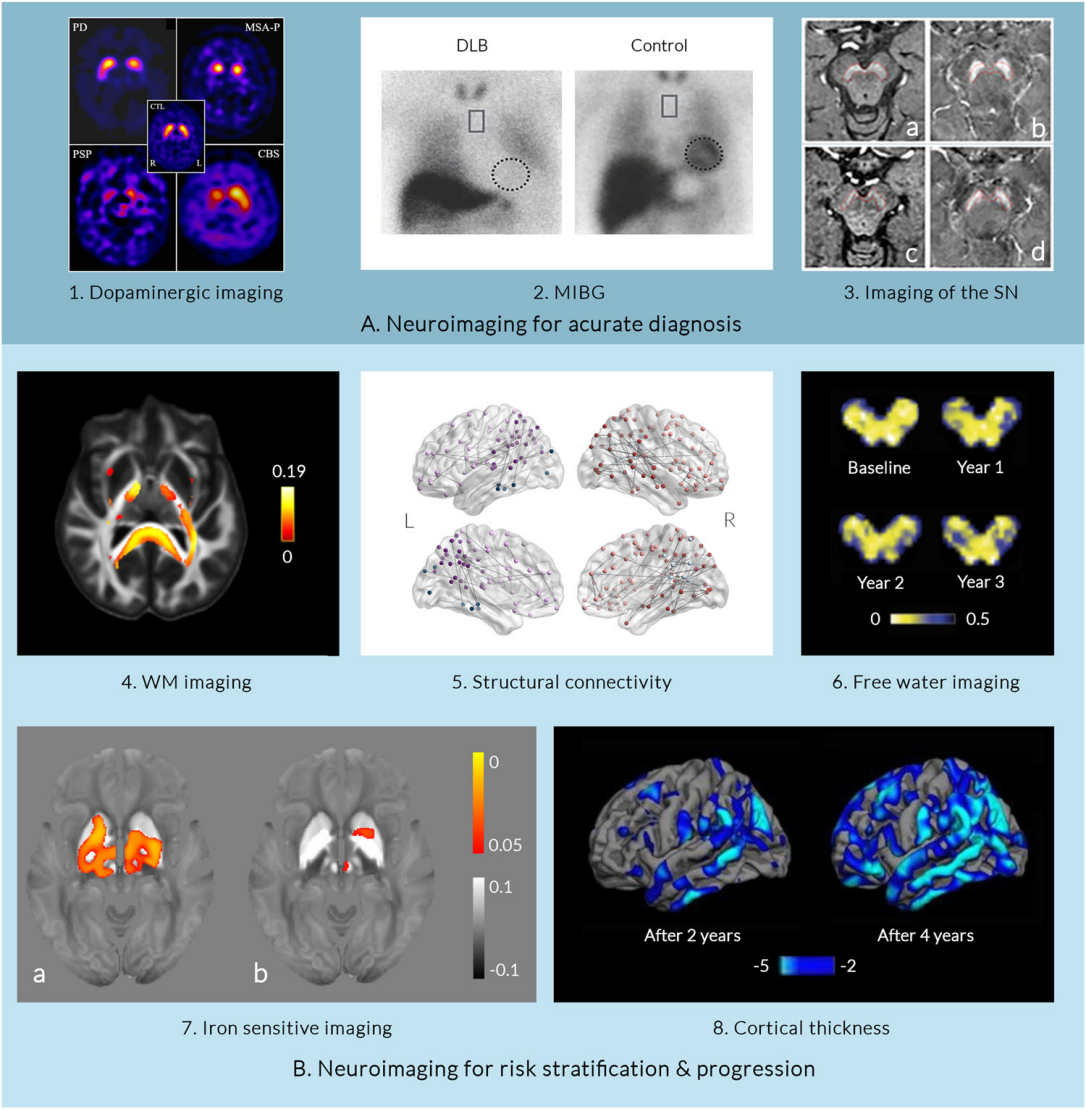
Emerging imaging measures in Parkinson’s disease. **A** Imaging measures for accurate diagnosis of Parkinson’s disease (PD) include: 1. *Dopaminergic imaging:* axial 123I-FP-CIT single-photon emission computed tomography (SPECT) scans for neuropathologically-confirmed degenerative parkinsonism; adapted from Nicastro et al.^21^ (Released under a Creative Commons Attribution-NonCommercial-NoDerivs 4.0 International license). 2. *Cardiac meta-iodobenzylguanidine (MIBG) scintigraphy:* Absent tracer uptake in the heart (dotted circle) in a patient with dementia with Lewy Bodies (DLB) compared to a control. Heart-to-mediastinum ratios (heart: dotted circles, upper mediastinum: rectangle) can be calculated. Adapted from ref. ^36^; 3. Imaging of the Substantia Nigra (SN): Neuromelanin-sensitive MRI (NM-MRI) to visualise SN atrophy, with earliest changes involving signal loss in nigrosome-1 territory, caudal and mediolateral portions of the SN. Loss of nigrosome-1 shown here in a PD patient in an NM-MRI image (c) and QSM image (d) compared to a control (a: NM-MRI, b: QSM). Adapted with permission from ref. ^42^ (Released under a Creative Commons Attribution-NonCommercial-NoDerivs 4.0 International license). **B** Neuroimaging measures for risk stratification and tracking disease progression in PD: 4. Imaging of White Matter: Fixel-based analysis shows reduced white matter fibre cross-section in PD patients who later developed poor outcomes over 3-year follow-up. Colour scale represents percentage reduction in fibre cross-section in PD patients with poor vs PD patients with good outcomes. Adapted from ref. ^142^; 5 Structural connectivity: Widespread reductions in structural connectivity, mostly in frontal and parietal-occipital connections, seen at baseline in PD patients who progress to poor outcomes within 3 years, from ref. ^142^; 6. Free water imaging: Longitudinal changes in free water within the SN seen in PD patients compared to controls. Loss of signal is seen in the midbrain of a PD patient over 4 years. Adapted with permissions^179^, (released under a Creative Commons Attribution-NonCommercial 4.0 International license) 7. Iron-sensitive imaging: QSM signal at baseline correlates with longitudinal worsening of (a)motor and (b)cognitive scores in PD patients over 3 years. Colour bar represents p-value family-wise error-corrected for multiple comparisons, grey scale represents susceptibility values. Adapted from ref. ^173^ 8. Cortical thickness: Cortical thinning patterns in patients with mild PD compared to controls after 2 and 4-year follow-up. Statistically-significant regions shown in blue, corrected for multiple comparisons using false discovery rate. Colour bar represents *t*-values. Adapted with permission from Fillippi et al.^123^(Released under a Creative Commons Attribution-NonCommercial-NoDerivs 4.0 International license). CBS Corticobasal syndrome, CTL Control, DLB Dementia with Lewy bodies, L Left; MCI Mild cognitive impairment, MIBG meta-iodobenzylguanidine MSA-P Multiple system atrophy parkinsonian type, NM-MRI Neuromelanin-sensitive MRI, PD Parkinson’s disease, PSP Progressive supranuclear palsy; QSM Quantitative susceptibility mapping, R Right; SN Substantia nigra.

PD and other parkinsonian disorders are characterised by loss of striatal dopaminergic neurons and synapses. Resultant reduction in dopaminergic activity can be assessed using Positron emission tomography (PET) and single-photon emission computed tomography (SPECT) (Fig. 2a). A 2017 meta-analysis of 142 studies of presynaptic DAT and SPECT imaging in PD found that across all ligands, the greatest reduction in uptake was observed in the putamen, particularly in posterior regions. Across all regions, ligands for vesicular monoamine transporter 2 (VMAT2, reflecting vesicular dopamine storage) were most sensitive at separating PD from controls, while those for aromatic amino acid decarboxylase (AADC, relating to dopamine synthesis) were least sensitive^19^. This likely reflects compensatory mechanisms to increase synaptic availability of dopamine^20^. Ligands measuring presynaptic processes may be more sensitive in PD due to relative preservation, or even upregulation of postsynaptic dopamine receptors early in the disease. Dopaminergic imaging may also have potential to detect neurodegeneration at premotor stages of PD, although with less sensitivity^21,22^ (Table 1), with reduced striatal uptake observed in idiopathic REM sleep behaviour disorder (RBD)^23^ and hyposmia^24^; and in differentiating PD from atypical^21^ and vascular parkinsonism^25^, with machine-learning-based approaches potentially able to distinguish these at the individual level^26^. However, whilst dopaminergic imaging techniques show some decreases in PET signal soon after diagnosis, these begin to plateau within five years’ of diagnosis, limiting their ability as markers of disease progression^6^.

**Table 1:** Sensitivity and specificity of biomarkers of dopamine dysfunction for early and accurate diagnosis of Parkinson’s disease (PD)

Biomarker	Application	Sensitivity [95% confidence intervals]	Specificity [95% confidence intervals]	Utility & Evidence
Dopaminergic imaging	Overall accuracy of clinical diagnosis of a parkinsonian syndrome^22^	Early: 79% [68–87%] Clinically established diagnosis: 97% [94–99%]	Early: 97% [84–99%] Clinically established diagnosis: 98% [91–100%]	Clinical & Research: Validated biomarker
	Distinguishing PD from vascular or drug induced parkinsonism^25^	86% [81–90%]	83% [68-93%]	Clinical: Validated biomarker
Neuromelanin-sensitive MRI (NM-MRI)	Distinguishing PD from controls^10^	89% [83–100%]^a^	83% [53–100%]^a^	Research: Validated biomarker
	Distinguishing PD from atypical Parkinsonian syndromes^54^	Locus coeruleus: MSA-P: 60%^b^ PSP: 63.3%%^b^ Lateral substantia nigra: PSP: 86.7%^b^	Locus coeruleus: MSA-P: 90%^b^ PSP: 92.2%^b^ Lateral substantia nigra: PSP: 76.9%^b^	Research: Further validation is needed
Dorsolateral nigral hyperintensity/loss of swallow-tail sign	Distinguishing PD from controls^10^	96% [76–100%]^a^	95% [79–100%]^a^	Clinical: Validated biomarker
Free water imaging for the SN	Distinguishing PD from controls^50^	Prodromal disease: 74.4%^b^ Newly diagnosed PD: 98%^b^	Prodromal disease: 70.8%^b^ Newly diagnosed PD: 75%^b^	Research: Validated biomarker
Iron sensitive imaging of the substantia nigra	Distinguishing PD from controls^63–79^^c^	QSM^63–77^: 86.9% [58.1–100%]^a^ R2^a^^63,68,77–79^: 65.1% [56%-81%]^a^	QSM^63–77^: 77.1% [45–91.3%]^a^ R2^a^^63,68,77–79^: 74.5% [52–85.7%]^a^	Research: Validated biomarker

*MSA-P* Multiple system atrophy with parkinsonism, *PD* Parkinson’s disease, *PSP* Progressive supranuclear palsy, *QSM* quantitative susceptibility mapping.

^a^Pooled sensitivities and specificities from meta-analyses. Range of results from individual studies in brackets.

^b^Confidence intervals not reported.

^c^Pooled sensitivities and specificities calculated from the cited studies using a bivariate hierarchical summary receiver operating characteristic model.

#### Imaging of cerebral glucose metabolism

Another PET-based approach is to quantitatively assess cerebral glucose metabolism as a marker for neuronal function, using the radiolabelled glucose analogue 2-deoxy-2-[18 F]fluoro-D-glucose (18F-FDG). The sensitivity of 18F-FDG PET to functional changes outside the striatum allows it to detect distinctive patterns of functional change in PD^27^, with greater utility for differential diagnosis from atypical Parkinsonism than dopaminergic PET^28^. Recent work has also described a pattern of cerebral glucose hypometabolism in iRBD associated with conversion to PD^29,30^, with occipital hypometabolism a key feature, although this application of FDG-PET requires further validation.

#### Cardiac meta-iodobenzylguanidine (MIBG) scintigraphy

MIBG scintigraphy provides a measure of cardiac sympathetic denervation. MIBG is an analogue of an adrenergic blocking agent that is actively taken-up into post-ganglionic presynaptic nerve endings of the adrenergic nervous system. When labelled with 123Iodine it can be visualised using cardiac scintigraphy, and quantified by comparing uptake in a region of interest over the heart with a control region over the mediastinum, providing the heart-mediastinum ratio (Fig. 2a).

Reduced cardiac MIBG uptake in PD was first shown in the 1990s^31^, and can discriminate PD from atypical parkinsonian disorders and mimics^32^ with over 85% sensitivity and specificity (Table 2)^33^. A recent prospective study of 288 people with de novo PD, showed that cardiac MIBG had higher diagnostic accuracy than DAT-SPECT imaging, with 92% sensitivity and 94% specificity^34^. In 2015, cardiac MIBG was included as a supportive element for diagnostic criteria for PD^35^ and in 2017, added to diagnostic criteria for DLB (dementia with Lewy bodies)^36^. There may also be a role for cardiac MIBG in defining distinct PD phenotypes: early studies suggested greatest loss of uptake in akinetic-rigid than tremor-dominant disease^37^. More recently, loss of cardiac MIBG uptake was linked with a “body first” as opposed to “brain first” phenotype, with associated loss of 11C-donepezil PET-CT signal, reflecting reduced cholinergic parasympathetic innervation^38^.

**Table 2:** Sensitivity and specificity of biomarkers of alpha-synuclein, and other biomarkers, for early and accurate diagnosis of Parkinson’s disease (PD)

Biomarker	Application	Sensitivity [95% confidence intervals]	Specificity [95% confidence intervals]	Utility & Evidence
Alpha-synuclein seed amplification assay (SAA)	Distinguishing PD from controls^11,101,103–105^	CSF^11^: 88% [82–93]^a^ Skin biopsy^101^: 75%^b^ Saliva^103^: 76% [66.1–85.9%] Submandibular gland^104^: 56.1%^b^ Plasma^105^: 94/6%	CSF^11^: 95% [92–97%]^a^ Skin biopsy^101^: 83%^b^ Saliva^103^: 94.4% [86.6–100%] Submandibular gland^104^: 92.9%^b^ Plasma^105^: 92.2%	Research and future clinical applications: Validated in CSF, further validation is needed for other samples
Neuron-derived exosomal alpha-synuclein	Distinguishing PD from controls^106^	82–85%	71-74%	Research: Further validation is needed
Other biomarkers				
Cardiac meta-iodobenzylguanidine (MIBG) scintigraphy	Distinguishing PD from controls^33^	88% [86–90%]^a^	85% [81–88%]^a^	Clinical & research: Validated biomarker
Imaging of cerebral glucose metabolism	Distinguishing PD from controls^27^	93% [84–100%]	93% [85–100%]	Research: Further validation needed
	Distinguishing PD from atypical Parkinsonian syndrome^28^	91.4% [76.5–100%]^a^	94.7% [75–98%]^a^	Research: Further validation needed

^a^Pooled sensitivities and specificities from meta-analyses. Range of results from individual studies in brackets.

^b^Confidence intervals not reported.

#### MRI techniques sensitive to substantia nigra changes

By the time motor symptoms present in PD, extensive loss of dopaminergic neurons in the substantia nigra (SN) has already occured^39^. Therefore, MRI techniques sensitive to related microstructural changes have potential as diagnostic biomarkers of PD.

The pigment neuromelanin (NM) is found at high concentrations in the SN and locus coeruleus (LC), where it is synthesised through polymerisation of dopamine-protein adducts^40^. Degeneration of SN neurons results in loss of NM and is detectable using neuromelanin-sensitive MRI (NM-MRI), which normally comprises a T1-weighted turbo spin echo sequence or magnetisation transfer-weighted gradient echo sequence (Fig. 2a). The NM signal is primarily due to the interaction between NM and the iron it chelates, and the influence this has on both T1-relaxation and magnetisation transfer effects^41^. NM-MRI signal loss in PD has been consistently demonstrated in the SN and LC^42–45^. In a 2021 meta-analysis, NM-MRI of the SN and LC could distinguish between PD and controls with a pooled sensitivity of 89% and specificity 83%^10^. NM-MRI may also have utility in detecting preclinical PD, as NM alterations are seen in iRBD^45,46^, although others have reported negative findings^47^. One study combining NM-MRI with fractional anisotropy measures was able to distinguish between iRBD and control with high diagnostic accuracy^48^. However, NM-MRI cannot be used to distinguish between PD and atypical Parkinsonism^49,50^, and is so far only sensitive at group level.

The loss of NM also leads to a reduction in dorsolateral nigral hyperintensity in nigrosome 1 of the substania nigra on susceptibility-weighted images, often referred to as loss of the swallow-tail sign. This is a robust biomarker, with a 2021 meta-analysis showing this can accurately -distinguish between PD and controls^10^ (Table 1).

Free water imaging, calculated from diffusion weighted MRI, separating the diffusion properties within tissue from those in the extracellular space, can also be used as a measure of SN dopaminergic neuronal degeneration in PD, where increases in extracellular space correspond to loss of neurons^51^. Free water content in the posterior SN can distinguish between PD and controls^52,53^, and incorporating information on free water content in the basal ganglia may help distinguish PD from atypical parkinsonism^54^. Posterior SN free water content also differs between PD and iRBD^55^.

Increased tissue iron content is seen in association with neurodegeneration in the SN in PD at postmortem^56^. Iron can cause cell stress and atrophy through toxic free reactive oxygen species, that in turn cause damage to DNA; affect mitochondrial function; and modify proteins though reactive aldehydes, leading to iron-mediated cell death, or ferroptosis^57^. In addition, excess iron promotes aggregation of alpha-synuclein fibrils^58^. Iron stored in ferritin is the primary contributor to paramagnetic signal in deep brain nuclei when using quantitative susceptibility mapping (QSM) and R2*. Accordingly, robust and consistent increases in iron-related signal in the PD SN are seen using QSM and R2*^56,59^. While NM loss is localised only to the SN and LC in PD, increases in brain tissue iron concentration in PD relative to controls are seen throughout the brain using QSM^60–62^, most commonly in the basal ganglia. A number of studies have used QSM^63–77^ or R2* values^63,68,77–79^ in the substantia nigra to discriminate between PD and healthy controls (Table 1) with QSM generally outperforming R2* in this context^80^. Approaches additionally considering QSM values outside of the SN have shown improved diagnostic accuracy between controls and PD^81^, as have approaches using QSM in combination with NM-MRI^70,82^ or to augment interpretation of the N1 sign^83^. As with NM-MRI and free water content, increased iron measured using QSM is also found in iRBD^74,84^. Crucially, increases in both iron^85^ and NM^86^ in the SN are observed across the lifespan during normal aging, including the period where PD typically manifests. As such, age will need to be accounted for when using these tools for diagnostic use.

### Diagnostic fluid biomarkers

The pathological hallmark of PD is the build-up of intracellular aggregates of alpha-synuclein, formed due to protein misfolding. Although total alpha-synuclein can be measured in body fluids such as CSF, values are heterogeneous, with overlap between patients and controls. This arises due to extremely low concentrations in CSF (with high levels in blood causing contamination), and vulnerability to factors such as time before storage and number of freeze-thaw cycles^87^. Measuring total alpha-synuclein is also unlikely to reflect disease status, as the smaller oligomeric species are thought to be the most damaging forms.

#### Seed amplification assays

A recent approach for detecting alpha-synuclein is based on the ability of alpha-synuclein seeds to cause monomeric alpha-synuclein to form oligomeric and then fibrillary forms. This technique was originally adapted by Green and colleagues from real time quaking induced conversion (RT-QuIC) assays used to detect prion protein^88^. For this assay, biological sample is added to a well containing monomeric alpha-synuclein and thioflavin T (ThT). If the sample contains pathological alpha-synuclein, it induces templating, misfolding and aggregation of the monomers, resulting in a high β-pleated sheet content of the protein, which binds ThT, resulting in increased fluorescence after several hours. A similar approach known as protein misfolding cyclic amplification was used by Soto and colleagues^89^. The common term alpha-synuclein seed amplification assay (SAA) is now used to apply to both approaches, which have since been replicated by other groups^90–92^. The assay has been made more streamlined and faster^91^, and is reproducible across laboratories^93^ with pooled sensitivity and specificity for CSF alpha-synuclein SAA across 22 studies, 88% and 95% respectively^11^ (Table 2). Alpha-synuclein SAA can also identify prodromal stages of synucleinopathy in iRBD^94,95^, with pooled sensitivity of 74% and specificity 93%^11^. Currently, most SAAs report a dichotomous positive or negative readout, but additional metrics such as lag time, maximum fluorescence, and time to 50% fluorescence can also be calculated, providing potential quantification. Faster kinetics and higher maximum fluorescence values are seen in DLB compared to PD^91,93^; and more rapid kinetics are seen for PD patients with poorer cognitive scores^96^. However, relationships with motor severity^92,97^ are not seen. A recent study of 1100 patients from the Parkinson’s Progression Marker Initiative (PPMI), including a large number with genetic variants, showed that alpha-synuclein SAA positivity varied, depending on genetic and clinical features^98^. Alpha-synuclein SAA positivity in PD was highest for patients with *GBA* mutations (95.9%), and lowest with *LRRK2* mutations (67.5%), consistent with previous studies^99,100^. Notably, RBD patients with hyposmia had higher levels of SAA positivity, which was also higher than DAT positivity, suggesting that alpha-synuclein SAA becomes abnormal before DAT imaging^98^.

However, obtaining CSF is relatively invasive, and not amenable to repeat sampling, prompting attempts for SAAs in other biological samples. Alpha-synuclein SAAs performed on skin biopsies have shown similar sensitivities and specificities as CSF^101^; whereas nasal brushing to access the olfactory mucosa has lower accuracy, and the sampling method requires a rigid scope and otolaryngologist to access the deep structures^102^. Other tissue suitable for SAA include submandibular glands^101^; saliva (albeit with lower sensitivity than CSF)^103^; and colonic biopsies^104^. More recently, immunoprecipitation-based RT-QUiC, detected pathogenic alpha-synuclein in serum, with high sensitivity and specificity in discriminating PD as well as DLB from controls^105^.

#### Extracellular vesicles

Another approach showing strong potential to provide useful diagnostic blood tests for PD is the quantification of proteins released from brain-derived extracellular vesicles (EVs)^106^. These can be released from any cell type and carry proteins, DNA, RNA and glycoconjugates. Critically, they can cross the blood-brain barrier, resulting in contents from neuronal cells being carried into the periphery where they can be more easily measured. A range of techniques can be used to isolate EVs, with L1CAM immunoprecipitation particularly effective for identifying neuronal EVs^107^. In a recent study of 275 PD patients and 144 controls, as well as atypical PD cohorts, neuronal EVs were isolated using L1CAM immunocapture, followed by mass spectrometry or electrochemiluminescence to measure proteins including alpha-synuclein and clusterin^106^: Compared with controls and other neurodegenerative conditions including MSA, levels of mean neuron-derived exosomal alpha-synuclein were two-fold higher in clinical PD and prodromal PD, with an AUC of 0.86 for separating patients with PD from controls^106^. An even higher AUC of 0.98 was found when combined with measures of clusterin, an exosome-associated protein^106^. Stuendl et al similarly purified EVs (although without enrichment for brain-derived EVs); quantified alpha-synuclein content using electrochemiluminescence; and showed that EV-derived alpha-synuclein was able to discriminate PD from controls (AUC 0.77) and had an inverse relationship with motor severity^108^. More recently Kluge et al isolated EVs from plasma using immunoblotting, dynamic light scattering and transmission electron microscopy, with L1CAM signals to confirm neuronal origins of the EVs. They then extracted and amplified pathological alpha-synuclein using an SAA, which was able to correctly identify all 30 PD samples^109^, although this has not yet been independently validated.

#### Omics

In contrast to hypothesis-driven searches for biomarkers, proteomics provide an alternative, data-driven approach. Several groups have recently applied proximity extension assays (PEA) to detect and quantify very large numbers of proteins simultaneously in biological samples, especially plasma. These are immunoassays, leveraging antibody-oligonucleotide protein binding for quantitative real-time polymerase chain reaction measurements. Using this approach, a number of proteins have emerged showing differences between PD patients and controls. CCL28 a protein bridging the innate and adaptive immune system, was increased in the CSF of patients with PD^110^. In a study using large-scale PEA of 276 proteins in oncology, cardiovascular and metabolism panels, dopa-decarboxylase (DDC), which catalyses the decarboxylation of dopa to dopamine, was also increased in the CSF of PD patients compared to controls and was the most significant protein in separating those groups^13^. Differences in DDC correlated with dopaminergic treatment, but were also seen in drug-naïve patients, showing that this was not a direct effect of treatment^13^.

DDC was also the top biomarker found in a PEA study in 48 PD and 347 controls using an extensive panel of 2493 proteins in CSF and 92 proteins in plasma^14^. Higher DDC levels correlated with worse cognitive scores and a higher risk of progressing to clinical LBD during follow-up. A suggested mechanism for this finding was a form of compensatory upregulation in patients with low dopamine levels. Other markers identified using PEA approaches in plasma include up-and down-regulation of inflammation markers in PD compared with controls; and lower levels of markers commonly linked with cardiovascular risk^111^.

An even more powerful approach in terms of potential scale of proteins that can be tested is aptamer-based technology^112^. This uses short single-stranded oligonucleotides that can bind with high specificity to proteins and small molecules, enabling highly multiplexed proteomics assays. In plasma samples from two separate cohorts, including a total of 311 patients with PD, 1129 proteins were measured with this type of aptamer-based platform^113^. Four proteins differentiated PD from controls: bone sialoprotein, osteomodulin, aminocyclase and growth hormone receptor. This aptamer-based approach can be used for large-scale biomarker discovery. A recent programme examining 4006 proteins from serum, CSF and brain tissue^114^, identified a signature of proteins more strongly associated with PD (with AUC ranging from 0.66 for serum, to 0.75 for brain tissue), including proteins from cytokine receptor interaction and complement pathways. Similarly, Abdi et al.^115^ examined 1300 proteins in serum in a PD cohort followed-up for up to 8 years, and revealed 4 proteins potentially discriminating between PD and controls with a relationship to longitudinal clinical progression. These were NOTCH1, which has a role in neuronal and glial proliferation; ALCAM, a cell adhesion molecule involved in axonal guidance and growth; DUS3, which regulates cell differentiation; and CD36, which is involved in mediating microglial pro-inflammatory responses.

The field is changing rapidly, with multiple techniques emerging in the last 5 years alone, and a number of fluid-based biomarkers already show promise both for clinical application and use in PD research. SAAs have good accuracy in discriminating controls from PD, with AUCs of up to 0.93^92,116^, and potentially even discriminating DLB from PD^105^. This is still below the diagnostic accuracy displayed by fluid biomarkers in Alzheimer’s disease. For example p-tau217 has been shown to distinguish between Alzheimer’s and controls with an AUC of 0.98^117^, although SAA accuracy may increase in the coming years. However, the gold-standard fluid required for SAA remains CSF, which means relatively invasive collection compared with plasma. This, combined with the lack of meaningful quantification may limit SAA usage to diagnosis, rather than as a marker of progression in PD. Omics show significant promise in elucidating information about PD disease mechanisms, and the data-driven approach could reveal new targets for future clinical trials or new assay development. For example DDC could distinguish between controls and synucleinopathies with an AUC of 0.89. However, such approaches are less likely to be useful clinically in of themselves due to lack of precisely-defined outcome measures and considerable overlap between disease groups. EVs show the greatest promise for future clinical application. They are accessible from blood, and early results indicate that EV-based assays display extremely high accuracy in distinguishing between PD and controls^109^. Approaches that combine several EV protein measures may be the most fruitful for the clinic and for trials, as these significantly increase AUCs for separating PD from other proteinopathies (to 0.98)^106^, and some studies suggest potential for meaningful quantification^108^.

### Staging systems leveraging diagnostic measures

Two biomarker-based staging systems have recently been proposed that leverage some of these methods. They are motivated by a need to consider PD biologically, rather than as a clinical syndrome, and follow a similar framework to the A/T/N approach established for Alzheimer’s^118^. This is a system that categorizes individuals based on the presence of specific disease-relevant biomarkers, with beta-amyloid (A, measured using CSF or amyloid PET), hyper-phosphorylated tau (T, measured using CSF p-tau or tau PET) and neurodegeneration (N, measured by atrophy on structural MRI, FDG PET or CSF total tau).

Both of the proposed systems for staging in PD are based on the concept that pathological alpha-synuclein aggregation is the fundamental pathological process defining PD; that dopaminergic dysfunction in the midbrain is eventually universally present; and move away from purely clinically-defined syndromes.

Simuni et al.^4^ propose a biological definition for neuronal alpha-synuclein disease, a unifying term that encompasses alpha-synucleinopathies such as PD and DLB. They define two key anchors: S, representing neuronal alpha-synuclein, measured using CSF SAA, with two levels S- or S + ; and D, representing midbrain dopaminergic neuronal degeneration, detected using DAT-SPECT imaging, defined as D- or D + . They also consider genetic status, particularly for fully penetrant genes such as *SNCA*. They propose a staging system that takes into account these anchors, and starts from Stage 0, preclinical disease, defined by the presence of fully penetrant *SNCA* variants. Stage 1 and beyond all require SAA positivity, and from stage 2B, evidence of dopaminergic dysfunction is also required, although this may be present at Stage 1B. Stage 1 is defined as no clinical signs or symptoms, with subtle signs being present at Stage 2 that are mainly non-motor in nature. Patients in Stages 3-6 show functional impairment “that can be driven by motor, cognitive or other non-motor clinical signs or symptoms”. Each stage from 3 onwards is defined respectively as slight, mild, moderate and severe. Patients currently undergoing clinical trials for neuroprotection at early stages are already in Stage 3, and motor functional impairment is not present before this stage. In effect, this proposed staging system will mean that patients can be identified and staged for recruitment into neuroprotective trials at even earlier stages of PD, including prodromal stages, at Stages 1 and 2 of the system.

Höglinger et al.^5^ similarly consider alpha-synuclein positivity using the alpha-synuclein SAA from CSF or skin biopsies, as well as neurodegeneration, measured with imaging including dopaminergic PET/SPECT. In addition, they consider genetic variants strongly linked with PD, including *SNCA*, *GBA*, and *LRRK2*.

Both approaches acknowledge that future versions will likely incorporate novel alpha-synuclein measures, and additional biomarkers reflecting co-pathology. Whilst designed primarily for PD, they allow for prodromal forms and other synucleinopathies such as DLB to be included in their framework. They have the potential to enable better biological characterisation and matching of clinical cohorts across studies, as well as stratification for clinical trials, and more targeted treatments; although they both currently lack a system to track mid and late stage progression.

## Biomarkers of disease progression

In addition to aiding precise and early diagnosis of PD, biomarkers of disease progression are needed for monitoring outcomes in the clinic and in clinical trials. Quantitative and sensitive markers of progression have the additional benefit of risk stratification by identifying individuals at risk of faster progression and poorer outcomes. Accurate risk stratification at the time of diagnosis will allow earlier and more targeted selection of patients for treatments and clinical trials.

### Imaging markers

#### Imaging grey matter integrity

Markers of grey matter atrophy, using voxel-based morphometry or cortical thickness analyses have been extensively investigated as potential biomarkers of disease progression in PD, but with conflicting results. Individual PD patients do not often show easily detectable grey matter loss at diagnosis, although at group level, PD patients show cortical thinning and reduced subcortical and cerebellar volume compared to controls^119,120^. They also accumulate more cortical and basal ganglia atrophy than age-matched controls at longitudinal follow-up^7,119,121^. However, measures of grey matter atrophy are less useful in differentiating between patients with PD with different disease stages or outcomes: some studies have shown greater volume loss within right thalamus, hippocampus and fronto-temporal regions in PD with mild cognitive impairment (MCI) compared to those with intact cognition^122,123^ but this was not replicated in a recent multicentre study that only found differences with behavioural symptoms but not cognition at 1 year follow-up^124^.

High inter-individual variability in cortical atrophy patterns in PD may account for the lack of group differences in case-control statistical approaches. Normative modelling, which maps individual patterns of variation from the expected norm, takes advantage of this heterogeneity and could be a more sensitive approach. In Alzheimer’s disease, the number of regions that are outliers in cortical atrophy correlate with poorer cognition and predict future conversion to dementia^125^. In PD and DLB, outlier counts are also higher in patients with dementia, and correlate with worse cognition^126^.

Another potentially more sensitive technique for assessing grey matter is to examine microstructure using diffusion weighted imaging. Higher mean diffusivity within the grey matter is thought to reflect damage to dendrites or cellular membranes causing less restricted water diffusion within the grey matter. In a region-of-interest analysis of the basal ganglia in the PPMI cohort, baseline mean diffusivity of the globus pallidus was associated with worsening motor severity, cognition, and total symptom burden after 4.5 years’ follow-up^127^. In the same cohort, using a different subset of patients and whole brain data with 2 year follow-up, increased mean diffusivity within frontal and temporal cortical regions were associated with faster decline in cognition and faster increases in non-motor, but not motor disease burden^128^.

#### Imaging white matter integrity, micro- and macro-structure

Cell and animal models of PD suggest that axonal changes precede neuronal loss in PD^129^. Therefore, measures sensitive to changes in white matter tracts are likely to be more sensitive in PD. Indeed, in a recent cross-sectional simulation of progression of structural alterations in 130 patients with PD, Park et al showed earlier white matter changes than cortical thickness loss^130^. Additionally, several studies using diffusion tensor imaging have shown changes in fractional anisotropy or mean diffusivity within the white matter, in the absence of grey matter changes in PD^131–133^. These white matter alterations increase with worsening cognitive decline, particularly within the corpus callosum^134^, and with motor severity^135^.

However, diffusion tensor imaging results have not been fully consistent: a longitudinal multicentre study of 423 newly diagnosed PD patients followed-up over 3 years showed no correlation of baseline, or longitudinal diffusion tensor-derived measures with subsequent cognitive impairment^136^, with negative results seen in another independent cohort^137^. A potential reason for inconsistency in diffusion tensor-derived metrics is that they cannot accurately model crossing white matter fibres, which represent the majority of white matter tracts in the human brain^138^.

Newer higher-order diffusion models have emerged with more robust estimations for crossing fibres, with promising results in PD. Fixel-based analysis, one such higher-order model, has revealed reduced white matter fibre cross-section in the corpus callosum with disease progression in PD^139^; and widespread reductions are seen at baseline and longitudinally in patients with PD and poor visuoperceptual function, who are at higher risk of dementia^140^. We have recently shown that fibre cross-section is reduced at baseline with up to 19% reduction amongst several white matter tracts in cognitively intact patients with PD who progress to poor outcomes within 3-year follow-up^141^ (Fig. 2b). Fibre-specific measures of white matter integrity may be useful biomarkers predicting outcomes in PD, although further validation of their ability to track disease progression is needed.

### Structural and functional connectivity

Approaches that consider structural and functional connections at network level provide additional metrics and even mechanistic insights, with structural connectivity of particular interest given early white matter involvement. Reductions in structural connectivity are seen in medication-naïve PD patients increasing with disease duration^142^ and preserved structural connectivity within frontal and cerebellar regions are associated with slower motor symptom progression^143^. Baseline reductions in structural connectivity also predict progression to poor cognitive outcomes at 3-year follow-up^141^ and in PD patients with MCI they predict progression to dementia within 5 years^144^, with interhemispheric frontal and parietal connections being most implicated in cognitive impairment^141,144,145^.

Changes in functional connectivity have also been shown using functional MRI, where functional connectivity between two regions is defined as the similarity (commonly Pearson correlation) between the BOLD signal from those regions-. Both increases and reductions in resting state functional connectivity have been reported in relation to disease severity in PD, with the most consistent findings including reduced connectivity within the posterior putamen; however there is still variability in results between studies, likely due to divergent design, cohorts, and analytical methods^146^. Task-related functional connectivity is also altered in PD. In a recent metanalysis of 39 studies of functional connectivity during motor tasks, patients with PD consistently showed reduction in activation of the posterior putamen, cerebellum, primary motor cortex and supplementary motor area, but also increased activation to cortical regions connected to the anterior putamen^147^. However, these changes were not correlated with disease motor severity^147^.

Functional connectivity of specific brain regions may be more relevant to disease progression: a recent study assessing resting state functional connectivity of the striatum showed differences in spatial connectivity patterns in patients with PD which related to motor severity and levodopa administration^148^. In addition, key changes in resting state functional connectivity have emerged across studies relating to cognitive decline in PD. These are an overall increase in local connectivity, with reduction in long-range functional connectivity^149^; a temporal shift towards a more segregated functional connectivity^150,151^; and an uncoupling of functional from underlying structural connectivity in transmodal (higher association) regions^152^.

These changes in resting-state and task-induced functional connectivity provide useful mechanistic insights, but may not be as sensitive as white matter imaging in predicting longitudinal progression in PD^141^. As in the case of grey matter imaging, individual variability may partly underlie this, and recent approaches that leverage individual heterogeneity can predict motor outcomes in PD with potential for individual-level predictions^153^. An alternative explanation for the somewhat heterogeneous functional connectivity changes would be variable compensatory changes in functional connectivity within motor and cognitive networks^154^. This is in keeping with the upregulation of cortical areas seen during motor tasks in PD^147^. Interindividual variability in compensatory mechanisms which in itself, varies during the course of the disease, could be contributing to the heterogeneity of functional changes in PD as well as to heterogeneity in disease severity. Experiments could be designed to specifically assess compensation: a recent study using an action selection task, showed increased selection-related activation of parietal-premotor cortex in PD with milder clinical phenotype compared to both controls (suggesting upregulation of normal activity) and to PD patients with worse motor disease (suggesting that increased activity in these regions may be related to milder motor symptoms)^155^. Quantifying compensatory mechanisms could be an important biomarker to predict clinical progression in PD.

#### Imaging cerebrovascular effects

Coexistent cerebrovascular disease is increasingly recognised as having a synergistic effect on synucleopathies^156^ and multiple studies have assessed imaging markers of cerebrovascular disease in relation to outcomes in PD. White matter hyperintensity burden is higher in patients with PD dementia and MCI compared to patients with intact cognition^157^;. Total white matter hyperintensity volume at baseline predicts cognitive decline, but not motor progression, after follow-up^158,159^. Higher burden of cerebral microbleeds^160^ and lacunes^161^ is also associated with poorer cognitive scores. But other markers of cerebrovascular disease such as enlarged perivascular spaces are less explored to-date. Concurrent multimodal assessment of different markers of cerebrovascular disease and how these relate to white matter imaging changes are needed to disentangle the contribution of neurodegenerative and cerebrovascular causes on white matter degeneration in PD.

#### Cardiac MIBG scintigraphy

Although useful for PD diagnosis, it is less clear whether cardiac MIBG has a role in measuring disease progression. Early studies suggested a relationship with motor severity, especially for the early heart-mediastinum ratio^32,37^, but other studies have not found this^162,163^. In part, this may relate to a ceiling effect reached early in the disease course^164^. More recently, a relationship with other biomarkers has emerged, with correlation between cardiac MIBG uptake and plasma alpha-synuclein measured using ELISA^165^; and phosphorylated alpha-synuclein in autonomic nerve bundles on skin biopsy^166^. Finally, cluster analyses have shown that early MIBG changes and cortical atrophy are linked to a more malignant PD phenotype with more rapid decline^167^.

#### Iron-sensitive imaging

NM-related signal, detected using NM-MRI, has consistently shown decreases over the disease course in PD^45,168,169^. While this is useful in detecting changes, a key limitation of NM-MRI is its specificity to NM-containing regions, namely the SN and LC, making it relatively insensitive to markers of progression that manifest in other anatomical locations. This is particularly an issue in later disease stages, when the vast majority of NM-containing neurons have degenerated and a ceiling effect is observed.

Tissue iron content in the basal ganglia outside of the SN measured using QSM and R2* in PD is associated with disease duration and severity of motor symptoms^60^ (Fig. 2b), although not as consistently as within the SN^170^. Increased magnetic susceptibility in hippocampal and cortical regions is associated with cognitive severity at a single timepoint^62,171^, and increased susceptibility in the nucleus basalis of Meynert is predictive of poorer cognitive performance after follow-up^172^. However, longitudinal studies examining changes in deep brain nuclei QSM over the disease course in PD demonstrate inconsistent results, with increases^173^, decreases^174,175^ and no change^172^ all reported. R2*, which is additively sensitive to a wider range of causes of tissue changes, including cell-count, shows more consistent longitudinal increases during PD than QSM^176,177^, as does free-water imaging of the SN^178^. This suggests that tissue microstructure changes over the disease course are likely to include additional factors rather than simply a gross increase in ferritin-bound tissue iron. Such tissue changes may involve alterations in myelination^179^, pathological protein accumulation^180^, or changes in concentrations of other magnetically-reactive metals such as calcium, copper, and magnesium^179^.

### CSF and plasma biomarkers

Plasma and CSF biomarkers that can predict or track disease progression are increasingly being developed. Given the core role of alpha-synuclein aggregation in PD, great attention has been paid to quantify alpha-synuclein using varied immunoassays including ELISA, xMAP or electrochemiluminescence. However, total alpha-synuclein in the CSF, does not show a consistent relationship with Lewy body pathology, or change over time in relation to motor or cognitive measures^8,9^, and quantification metrics of the alpha-synuclein SAA are not yet validated or robust across different laboratories.

In contrast, CSF biomarkers of Alzheimer’s pathology at baseline, specifically higher CSF phosphorylated tau (p-tau), lower CSF abeta-42 and lower ratio of abeta-42 to total tau are all associated with worsening motor and cognitive performance at 3-year follow-up^181,182^. This is consistent with ex-vivo evidence for coexistent beta-amyloid and tau pathology in PD^2^. Plasma p-tau levels have not been as successful in predicting outcomes in PD, although they correlate well with CSF p-tau levels and are established in Alzheimer’s as a biomarker of tau and beta-amyloid pathology: levels of phosphorylated tau at threonine 181 (p-tau181) are not increased in PD patients who progress to dementia^141,183^. Alternative targets such as p-tau at threonine 217 (p-tau217) have been recently developed, showing better accuracy in Alzheimer’s^184^ and may be more relevant to poor cognitive outcomes in PD. Importantly, these biomarkers can now be reliably measured in blood.

Neurofilament light chain (NFL), a marker of underlying axonal damage, can be reliably quantified using immunoassay in both CSF and plasma, even at low concentrations, and has been shown across several cohorts to correlate with poorer outcomes: CSF NFL is increased in PD patients with cognitive impairment^185^ and higher plasma NFL values predict subsequent MCI and dementia^141,183,186^. However, NFL is a non-specific marker and rises in relation to white matter lesions across inflammatory and other degenerative conditions^187,188^, and with age, which may limit its use in clinical settings where comorbidities cannot be controlled. Plasma NFL has recently shown clinical utility in differentiating PD from atypical Parkinsonian disorders^189^.

Some of these markers can be combined in multiplex assays, improving the efficiency of testing, with reduced time and cost, and avoiding the same sample being handled multiple times. For example, the Simoa tetraplex panel provides information on NFL, GFAP, tau and UCHL-1. In a study comparing 29 PD patients and 30 controls, no difference was seen between groups, but a relationship between NFL and GFAP was seen for motor severity^190^.

Biomarkers of lysosomal and inflammatory pathology are also starting to be explored in PD as markers of poor outcomes. In the PPMI cohort, amongst patients with sporadic PD (negative for *GBA* mutations), patients with higher CSF glucocerebrosidase-to-sphingomyelin ratios showed faster cognitive decline over 3 years, although the ratio did not show significant longitudinal changes^191^. In another cross-sectional cohort, reduced glucocerebrosidase activity was seen in patients with more advanced motor disease (together with lower cathepsin D activity) and correlated with worse cognition^192^.

Increased inflammatory cytokines including interleukins 6, 4 and 8, C-reactive protein, tumour necrosis factor α, and transforming growth factor levels are correlated with worse motor function, whilst worse cognition is reported with higher levels of interleukins 6, 2, 8, 17, tumour necrosis factor α, and C-reactive protein in several studies^193^. However, significant heterogeneity in study design, measures of inflammatory biomarkers and analysis techniques^193^, makes evaluation of inflammatory markers compared to other markers of progression in PD difficult, and their clinical usefulness remains uncertain.

### Clinical markers of progression

Older age, male sex, higher baseline motor scores and worse baseline cognition have all been linked to worse motor and cognitive outcomes at longitudinal follow-up^141,182,194–196^ and have been integrated with sleep disorder, presence of depression and various genetic, imaging or CSF biomarkers into clinical predictive scores^182,196^. Increasing evidence also implicates visual system dysfunction as an early and predictive biomarker of poor outcomes in PD. The visual system is affected in PD, with worse performance of affected individuals across a range of visual tasks: spatial navigation, line orientation, visual rotation, facial recognition, contrast sensitivity, colour vision^197^. In a large-scale population study, patients with PD showed worse visual acuity than age-matched unaffected individuals^198^. Changes within the visual system in PD are even seen in the retina, with loss of dopamine in post-mortem and animal models^199^ and several studies showing thinning of ganglion cell and inner plexiform layers in PD patients^200^; with thinning correlating with poorer cognitive scores^201,202^, and seen years before clinical presentation^203^, albeit with small effect sizes. Although the whole of the visual system is affected in PD, higher-order visual tasks, rather than retinal changes, are stronger predictors of poor outcomes^202^ and, in predictive cross-sectional modelling, these occur before retinal changes in the sequence of progression to PD dementia^204^.

## Biomarkers for precise treatment

With novel treatments emerging for neurodegenerative diseases that target specific protein aggregations, stratification of subgroups enriched for specific targets will be crucial for clinical trials of novel interventions; and biomarkers revealing evidence of target engagement will be needed as outcome measures in these trials. Ultimately, disease modifying treatment in PD is likely to be individualised, with patients receiving the combination of therapies that best-matches their precise pathological phenotype, targeting not only alpha-synuclein but potentially amyloid and tau co-pathology, cerebrovascular disease or neuroinflammation. Similar pathological processes with prominent alpha-synuclein accumulation underlie PD, PD dementia and DLB making them challenging to distinguish using biomarkers. Instead, they should be considered a spectrum of disorders, separated by the timing of the onset of dementia. They can each involve differing combinations of underlying pathological processes. Robust and accurate measurements of these underlying pathological accumulations will be a critical part of applying new interventions in PD, as well as other synucleinopathies.

### Measures of underlying alpha-synuclein pathology

As the characteristic neuropathological hallmark of PD, alpha-synuclein would be expected to be detected in almost all patients. However, its presence alone does not explain progression to dementia, and total measured CSF alpha-synuclein does not correlate with disease progression or severity^8,9^. Serum and plasma alpha-synuclein immunoassays are even less successful, with reports of both higher, lower and no difference in PD compared to controls^205^. This overlap between groups may be due to alpha-synuclein occurring as multiple different species, and therefore greater precision in identifying relevant species may provide greater sensitivity. CSF oligomeric alpha-synuclein and oligomeric/total alpha-synuclein ratio, for example is increased in PD and associated with worsening motor signs, in particular in postural-instability PD subtypes^206^ although specificity remains low^205^. Higher oligomeric but not total-alpha-synuclein was also seen in the serum of PD patients compared to Alzheimer’s and controls in a small study^207^. Phosphorylated alpha-synuclein is similarly increased in plasma and red blood cells of PD patients compared to controls^205^. The alpha-synuclein SAAs, using CSF or more accessible fluids such as plasma, are likely to be important in stratification for trials and for targeted treatment, with potential for monitoring disease progression as quantification metrics become more reproducible.

An important gap in current efforts to image pathological protein accumulation in PD is the lack of a validated PET radiotracer for alpha-synuclein, restricting both diagnosis and assessment of disease progression. Development of potential tracers is hindered by several factors. The absolute concentration of alpha-synuclein aggregates within the brain is far lower than that of tau or beta-amyloid, and alpha-synuclein aggregates are often co-localised with tau and beta-amyloid aggregates, which are structurally similar due to the high number of beta-pleated sheets. This means any alpha-synuclein tracer would need to have extremely high affinity and selectivity for its target. Additionally, alpha-synuclein inclusions are intracellular, meaning tracers would need to cross both the blood brain barrier and the cell membrane. Finally, a lack of reliable assays restricts evaluation of candidate compounds^208^. Despite this, there have been promising recent reports of the use of a PET tracer, 18F-F0502B, with high selectivity for alpha-synuclein over tau and beta-amyloid, and displaying preferential uptake in non-human primate models of PD over controls^209^. Another potential PET tracer, 18F-ACI-12589, also with high alpha-synuclein selectivity, was recently tested in-vivo in humans, and was able to successfully discriminate MSA from controls, Alzheimer’s and progressive supranuclear palsy, although displayed relatively low uptake in PD^210^. If successful, an alpha-synuclein tracer will be transformative in the field. Firstly, it may reveal differing patterns of alpha-synuclein deposition that could aid in the differential diagnosis of PD, PDD and DLB, as well as atypical Parkinsonian syndromes, potentially in combination with amyloid and tau PET. Secondly, it would allow in-vivo modelling of the spatiotemporal dynamics of alpha-synuclein spread in synucleinopathies in large cohorts, how this varies between individuals, and how it is associated with the development of different symptoms. Finally, it could provide evidence of target engagement in clinical trials, in the way that beta-amyloid tracers showed unequivocally for anti-amyloid therapies^17^.

### Measures of beta-amyloid and tau co-existent pathology

The development of [11 C] Pittsburgh compound-B (11C-PIB) combined with PET imaging allowed visualisation of beta-amyloid in vivo, and beta-amyloid PET positivity is now a key biomarker for Alzheimer’s disease, with other radioligands including [18 F] florbetapen and [18 F] florbetapir since developed. Early studies of 11C-PIB uptake in PD suggested very little difference from controls^211,212^, and in a meta-analysis, while high beta-amyloid positivity was seen for DLB and PDD compared to controls, the positivity rate in PD was actually lower than in controls^213^. Interestingly, however, several studies have reported associations between increased beta-amyloid burden and poorer cognitive ability in PD, even at very low levels^214,215^, with increased beta-amyloid burden in PD predicting poorer future cognition^216^ and more rapid progression^217^.

In-vivo PET imaging of tau binding is also possible using radiotracers such as 18F-AV-1451, 18F-FDDNP and, more recently, 18F-PI-2620 and 18F-MK-6240. A recent meta-analysis found minimal differences between controls and PD^218^, and while some studies have reported decreased tau uptake in the SN in PD, this is due to off-target binding of NM by 18F-AV-1451. Second generation tau tracers do not display this off-target binding^219^, and when combined across studies, increased tau uptake is found in inferior temporal cortex in PD compared with controls, entorhinal cortex in PD with cognitive impairment compared with PD without cognitive impairment, and inferior temporal and occipital cortex in DLB compared to PD^218^. Like beta-amyloid, tau burden is associated with cognitive severity in PD^220,221^.

Plasma biomarkers of amyloid and tau pathology are becoming established in clinical practice and show excellent correlation with PET markers and underlying Alzheimer’s pathology^12^. In a small study of PD patients, fluid markers have also been concordant with PET imaging^222^, although their sensitivity and specificity in identifying underlying beta-amyloid and tau pathology in PD has not yet been shown. Combined methods that characterise both beta-amyloid and alpha-synuclein aggregates in the same sample may be even more informative. For example, optical single-molecule imaging methods can assess aggregates with intermolecular beta-sheet structure and in a small number of PD patients showed increased large aggregates compared with controls, with higher alpha-synuclein and lower beta-amyloid^223^.

It is perhaps counter-intuitive that the incidence and extent of beta-amyloid and tau pathology on imaging is so low in PD, when a large proportion of PDD patients show both tau and amyloid pathology at postmortem^181^. However, there is evidence that alpha-synuclein may inhibit beta-amyloid plaque formation^224^, while aggregated forms of alpha-synuclein promote the aggregation of tau^225^. Taken together, this suggests that beta-amyloid formation is inhibited in PD, but that tau aggregation, and its associated cognitive deficits^226^, are present even in PD patients with low beta-amyloid burden^220,221^. There may be a shorter asymptomatic phase of beta-amyloid deposition in presymptomatic PD dementia compared to Alzheimer’s disease^227^, which could include a relatively accelerated deposition of beta-amyloid and tau.

### Markers of mitochondrial sub-types of PD

It is well-established that mitochondrial dysfunction contributes to the pathogenesis of PD: cell models of PD include those using mitochondrial toxins^228^; exposure to environmental agents that target mitochondrial respiratory chain complexes is linked to higher risk of PD^229^; mitochondrial genetic defects are seen in post-mortem PD brain^230^ and variations in pathways controlling mitochondrial DNA increase the risk of PD. Subgroups of *LRRK2* mutations (one of the commonest genetic forms of PD) are thought to relate to mitochondrial dysfunction^231^. However, previous assays required live cells to detect mitochondrial dysfunction, which are not easily accessible in clinical practice or scalable for large cohorts. Recently, Qi et al described a blood-based marker of mitochondrial dysfunction using a PCR-based assay^232^. The authors were able to show increased damage to mitochondrial DNA in both idiopathic PD and in patients carrying the *G2019S LRRK2* mutation, and found that mitochondrial DNA damage correlated with LRRK2 kinase activity. This approach could allow precision-targeting of PD subtypes with mitochondrial dysfunction, as well as monitoring of LRRK2 inhibition in trials.

### Imaging of underlying neuroinflammation

Alongside plasma and CSF biomarkers of inflammation, which are not specific to central inflammation, PET imaging of neuroinflammatory processes may offer another avenue for stratification for treatment targeting inflammation, or monitoring disease in PD^233^. For example, the radioligand 11 C(R)PK11195 binds to the mitochondrial translocator protein, TSPO, which is preferentially expressed in activated microglia. Increased 11 C(R)PK11195 binding has been reported in temporal, parietal and occipital cortices in PD compared to controls, and even more strongly in PDD; the extent of binding shows an inverse relationship with cognitive ability^234^. However, other studies have found increased binding in the SN and putamen, but no differences in cortical regions between PD and controls^235^. Increased 11 C(R)PK11195 binding may even relate to preserved cognition and white matter integrity in PD^236^. However, the assumption that TSPO PET accurately reflects activated microglia in humans is based on the observation of increased TSPO expression seen in mice^237^. A recent meta-analysis of mouse datasets confirmed TSPO upregulation in mice, but increased TSPO expression was not seen in human macrophages^238^. The authors then directly examined TSPO expression in a range of mammalian species and showed that while it is increased in rodents, it is not increased in other mammals, including humans. They further compared microglial TSPO expression in neurodegenerative and neuroinflammatory diseases (Alzheimer’s, amyotrophic lateral sclerosis and multiple sclerosis), and in mouse models of these conditions. They showed that although TSPO expression increased significantly in the mouse models of each condition, it did not increase in humans. The authors suggest that their findings transform how TSPO PET signal is interpreted, and that it reflects local cell density than microglial activation in humans. As such, further work is needed to validate PET imaging of neuroinflammation as a biomarker in PD.

## Future directions

### Multimodal approaches within the brain and beyond

Methods that combine information across modalities may be more sensitive in tracking disease progression and predicting outcomes. These include approaches that combine information from both structural and functional organisational brain changes. The relationship between structural and functional connectivity is altered in PD, with less close coupling, particularly in PD patients at higher risk of dementia^152^ and those with visual hallucinations^239^. Another technique relates the effects of structural brain network alterations to brain function using network control theory. This approach can predict electrophysiological neural responses after stimulation in patients with epilepsy^240^ and response to non-invasive transcranial magnetic stimulation during language tasks^241^. In PD, recent applications of this framework provided insights into mechanisms of visual hallucinations^242^. Such approaches have potential to discriminate disease stages, predict progression as well as effects of brain activation on brain function^243^.

Multimodal information from wider body systems may even have promise as markers of disease progression in PD. In a large recent longitudinal study of healthy aging, in over 1200 healthy people using seven body and three brain modalities, Tian et al showed that body aging phenotypes, particularly of the cardiovascular system, preceded brain measures^244^. Attempts to incorporate information from other body systems in PD diagnosis are already underway. For example, for the cardiovascular system, MIBG is now an established supportive biomarker of PD. More recently data from the gastrointestinal system have been used. Work examining changes in the gut microbiome in PD patients revealed a potentially different microbiome structure compared to controls, with depletion of bacteria important for short-chain fatty-acid production^245^, and two bacteria species possibly related to disease severity^246^. As PD is a multisystem disorder with a long prodromal phase affecting autonomic, bowel, bladder and other systems, incorporating multimodal information from the wider body could be particularly informative for earlier diagnosis, and as a measure of progression.

### MRI quantification for information about tissue structure

Multiparametric quantitative MRI provides parametric maps of biophysical tissue properties that reflect the microenvironment of the underlying tissue, aspiring to provide “in vivo histology” of the human brain. Of these multiparametric maps, R2*is already relatively well-established^59,124,176,177^. However, other sequences provide additional information likely to be relevant in PD. The longitudinal relaxation rate (R1), which is sensitive to myelin, water content, and iron concentration is organised in specific spatial gradients in the human putamen and caudate and changes with age^247^, but has not yet been examined in PD. In addition to assessing individual signals (i.e. R1, R2*, etc), regional changes in the ratio of these signals may also provide useful information. In 99 patients with early-stage PD from the PPMI dataset, decreased T1w/T2w in the posterior putamen correlated with contralateral motor signs and uptake on DAT scans^247^. More recently the R1/R2* relaxivity ratio has been proposed as a signal sensitive to iron mobilisation capacity within human brain tissue, rather than just iron deposition^248^. This may have particular application at very early stages of PD when iron homeostasis could be affected prior to significant iron deposition.

Magnetic resonance electrical properties tomography (also called quantitative conductivity mapping) can be used to measure tissue conductivity and permittivity, reflecting changes in the ionic content of tissues. Although still in development and with several algorithms proposed, this approach has been recently successfully applied to brain tumours^249^ and in Alzheimer’s shows a relationship with cognitive severity^250^ and may have applications in PD.

Recently, PET ligands binding to synaptic vesicle 2a (SV2a), such as 11C-UCB-J, have been developed, providing a in vivo marker of synaptic density, thought to be a key pathomechanism in PD and PD progression to dementia^251^. Studies of 11C-UCB-J show promising early results, and have been successfully used to demonstrate synaptic reduction in preclinical models of PD^252^.

### Increasing signal-to-noise: Ultra-high field MRI

Technical advancements in neuroimaging leading to ultra-high field MRI, increase signal-to-noise ratios providing improved spatial resolution and tissue contrast. Ultra-high field MRI at 7 Tesla (7T) can identify small subcortical structures such as the SN sub-regions and locus coeruleus^253,254^. Although study numbers to date are low, 7T-derived measures of the SN show potential as a biomarker in PD: loss of the SN’s normally smooth lateral boundary is seen in PD^255^, with reduction in SN volume^256^ and lower fractional anisotropy^257^ compared to controls. SN volume was also related to longer disease duration and worse motor severity in a small cohort of 32 patients^256^.

Locus coeruleus integrity is also affected in PD, with increased neuromelanin signal in 78 patients with early PD compared to 36 controls^258^ whilst reduced T1 signal relates to worse cognition^259^. 7T MRI of these small subcortical structures and their connectivity patterns could potentially be useful in diagnosis, risk stratifying and predicting response to treatment, particularly response to DBS where 7T MRI has already been applied^260^. The sub-millimetre spatial resolution of ultra-high field MRI allows it to detect changes in microstructure and functional activity within cortical layers^261^. This may have application in PD, based on animal models that suggest early and preferential involvement of deep layers^262^. In the coming years, spatial resolution of MRI will likely reach 200μm with 11.7T and 14T MRI currently under development^263,264^; this will allow the visualisation of myelination differences within the cerebellum and the true separation of BOLD signals from all cortical layers as well as the quantification of brain metabolites including GABA via MR spectroscopy^264^. This will open new avenues for research into mechanistic understanding of PD and potential new treatment targets.

## Conclusions

We are moving from a conception of PD as a clinical syndrome, and instead adopting frameworks with precise biological definitions. It is important to note that most biomarker studies to date are based on analyses of group information, rather than at the level of the individual patient. How they can be applied at the individual level, as part of proposed staging systems and in clinical practice, will require further validation and investigation. Interpretation of both imaging and fluid biomarkers needs to be done in the context of clinical information; and for these to be useful in wider settings, we will require standardisation of sample collection and analysis pipelines and in many cases consideration of cost. Despite these challenges, by providing information about underlying pathological processes, imaging and fluid biomarkers bring important opportunities to enhance diagnostic frameworks, enable early diagnosis, capture underlying causes of heterogeneity of PD, and quantitatively track disease progression. In this way they ultimately provide hope for new interventions to slow the progression of PD.

## References

[1] Dorsey ER (2018). Global, regional, and national burden of Parkinson’s disease, 1990–2016: a systematic analysis for the Global Burden of Disease Study 2016. Lancet Neurol..

[2] Irwin DJ (2017). Neuropathological and genetic correlates of survival and dementia onset in synucleinopathies: a retrospective analysis. Lancet Neurol..

[3] Vijiaratnam N, Simuni T, Bandmann O, Morris HR, Foltynie T (2021). Progress towards therapies for disease modification in Parkinson’s disease. Lancet Neurol..

[4] Simuni, T. et al. Biological Definition of Neuronal alpha-Synuclein Disease: Towards an Integrated Staging System for Research 10.1016/S1474-4422(23)00405-2 (2024). 10.1016/S1474-4422(23)00405-2 38267190

[5] Höglinger GU (2024). A biological classification of Parkinson’s disease: the SynNeurGe research diagnostic criteria. Lancet Neurol..

[6] Simuni T (2018). Longitudinal Change of Clinical and Biological Measures in Early Parkinson’s Disease: Parkinson’s Progression Markers Initiative Cohort. Mov. Disord. J. Mov. Disord. Soc..

[7] Mollenhauer B (2016). Monitoring of 30 marker candidates in early Parkinson disease as progression markers. Neurology.

[8] Mollenhauer B (2017). Longitudinal CSF biomarkers in patients with early Parkinson disease and healthy controls. Neurology.

[9] Hall S (2016). Longitudinal Measurements of Cerebrospinal Fluid Biomarkers in Parkinson’s Disease. Mov. Disord..

[10] Cho SJ (2021). Diagnostic performance of neuromelanin-sensitive magnetic resonance imaging for patients with Parkinson’s disease and factor analysis for its heterogeneity: a systematic review and meta-analysis. Eur. Radiol..

[11] Grossauer A (2023). α-Synuclein Seed Amplification Assays in the Diagnosis of Synucleinopathies Using Cerebrospinal Fluid—A Systematic Review and Meta-Analysis. Mov. Disord. Clin. Pract..

[12] Palmqvist S (2015). Detailed comparison of amyloid PET and CSF biomarkers for identifying early Alzheimer disease. Neurology.

[13] Paslawski W (2023). Large-scale proximity extension assay reveals CSF midkine and DOPA decarboxylase as supportive diagnostic biomarkers for Parkinson’s disease. Transl. Neurodegener..

[14] Pereira JB (2023). DOPA decarboxylase is an emerging biomarker for Parkinsonian disorders including preclinical Lewy body disease. Nat. Aging.

[15] Del Campo M (2023). CSF proteome profiling reveals biomarkers to discriminate dementia with Lewy bodies from Alzheimer´s disease. Nat. Commun..

[16] Cummings J (2023). Alzheimer’s disease drug development pipeline: 2023. Alzheimers Dement. N. Y. N..

[17] van Dyck CH (2023). Lecanemab in Early Alzheimer’s Disease. N. Engl. J. Med..

[18] Jiang Y (2022). Preclinical and randomized clinical evaluation of the p38α kinase inhibitor neflamapimod for basal forebrain cholinergic degeneration. Nat. Commun..

[19] Kaasinen V, Vahlberg T (2017). Striatal dopamine in Parkinson disease: A meta-analysis of imaging studies. Ann. Neurol..

[20] Ribeiro MJ (2002). Dopaminergic function and dopamine transporter binding assessed with positron emission tomography in Parkinson disease. Arch. Neurol..

[21] Nicastro N, Nencha U, Burkhard PR, Garibotto V (2023). Dopaminergic imaging in degenerative parkinsonisms, an established clinical diagnostic tool. J. neurochemistry.

[22] de la Fuente-Fernández R (2012). Role of DaTSCAN and clinical diagnosis in Parkinson disease. Neurology.

[23] Arnaldi D (2021). Dopaminergic imaging and clinical predictors for phenoconversion of REM sleep behaviour disorder. Brain: a J. Neurol..

[24] Jennings D (2017). Conversion to Parkinson disease in the PARS hyposmic and dopamine transporter-deficit prodromal cohort. JAMA Neurol..

[25] Brigo F, Matinella A, Erro R, Tinazzi M (2014). [^123^I]FP-CIT SPECT (DaTSCAN) may be a useful tool to differentiate between Parkinson’s disease and vascular or drug-induced parkinsonisms: a meta-analysis. Eur. J. Neurol..

[26] Chien C-Y, Hsu S-W, Lee T-L, Sung P-S, Lin C-C (2020). Using Artificial Neural Network to Discriminate Parkinson’s Disease from Other Parkinsonisms by Focusing on Putamen of Dopamine Transporter SPECT Images. Biomedicines.

[27] Brajkovic L (2017). The utility of FDG-PET in the differential diagnosis of Parkinsonism. Neurological Res..

[28] Meyer PT, Frings L, Rucker G, Hellwig S (2017). (18)F-FDG PET in Parkinsonism: Differential diagnosis and evaluation of cognitive impairment. J. Nucl. Med.: Off. Publ., Soc. Nucl. Med..

[29] Mattioli P (2023). Derivation and validation of a phenoconversion-related pattern in idiopathic rapid eye movement behavior disorder. Mov. Disord.: Off. J. Mov. Disord. Soc..

[30] Orso B (2023). Validation of the REM behaviour disorder phenoconversion-related pattern in an independent cohort. Neurological Sci.: Off. J. Ital. Neurological Soc. Ital. Soc. Clin. Neurophysiol..

[31] Yoshida M, Matsubara S, Tada A (1996). Decreased accumulation of 123I-metaiodobenzylguanidine myocardial scintigraphy in Parkinson’s disease. Shinkei Naika.

[32] Orimo S, Ozawa E, Nakade S, Sugimoto T, Mizusawa H (1999). 123I-metaiodobenzylguanidine myocardial scintigraphy in Parkinson’s disease. J. Neurol. Neurosurg. Psych..

[33] Treglia G (2012). MIBG scintigraphy in differential diagnosis of Parkinsonism: a meta-analysis. Clin. Auton. Res. J. Clin. Auton. Res. Soc..

[34] De Feo MS (2023). Role of Functional Neuroimaging with 123I-MIBG and 123I-FP-CIT in De Novo Parkinson’s Disease: A Multicenter Study. Life.

[35] Postuma RB (2015). MDS clinical diagnostic criteria for Parkinson’s disease. Mov. Disord..

[36] McKeith IG (2017). Diagnosis and management of dementia with Lewy bodies. Neurology.

[37] Saiki S (2004). Cardiac 123I-MIBG scintigraphy can assess the disease severity and phenotype of PD. J. Neurol. Sci..

[38] Horsager J (2020). Brain-first versus body-first Parkinson’s disease: a multimodal imaging case-control study. Brain J. Neurol..

[39] Cheng HC, Ulane CM, Burke RE (2010). Clinical progression in Parkinson disease and the neurobiology of axons. Ann. Neurol..

[40] Zucca FA (2017). Interactions of iron, dopamine and neuromelanin pathways in brain aging and Parkinson’s disease. Prog. Neurobiol..

[41] Trujillo P (2017). Contrast mechanisms associated with neuromelanin-MRI. Magn. Reson. Med..

[42] He N, Chen Y, LeWitt PA, Yan F, Haacke EM (2023). Application of neuromelanin MR imaging in Parkinson disease. J. Magn. Reson. Imaging.: JMRI.

[43] Hwang KS, Langley J, Tripathi R, Hu XP, Huddleston DE (2023). In vivo detection of substantia nigra and locus coeruleus volume loss in Parkinson’s disease using neuromelanin-sensitive MRI: Replication in two cohorts. PLoS One.

[44] Xing Y (2022). Neuromelanin-MRI to quantify and track nigral depigmentation in Parkinson’s disease: A multicenter longitudinal study using template-based standardized analysis. Mov. Disord..

[45] Biondetti E (2021). The spatiotemporal changes in dopamine, neuromelanin and iron characterizing Parkinson’s disease. Brain: a J. Neurol..

[46] Gaurav R (2022). Deep learning-based neuromelanin MRI changes of isolated REM sleep behavior disorder. Mov. Disord.: Off. J. Mov. Disord. Soc..

[47] Knudsen K (2018). In-vivo staging of pathology in REM sleep behaviour disorder: a multimodality imaging case-control study. Lancet Neurol..

[48] Pyatigorskaya, N. et al. Magnetic Resonance Imaging Biomarkers to Assess Substantia Nigra Damage in Idiopathic Rapid Eye Movement Sleep Behavior Disorder. *Sleep* **40**, zsx149 (2017). 10.1093/sleep/zsx149 28958075

[49] Kashihara K, Shinya T, Higaki F (2011). Reduction of neuromelanin-positive nigral volume in patients with MSA, PSP and CBD. Intern. Med..

[50] Ohtsuka C (2014). Differentiation of early-stage parkinsonisms using neuromelanin-sensitive magnetic resonance imaging. Parkinsonism Relat. Disord..

[51] Pasternak O (2012). Excessive extracellular volume reveals a neurodegenerative pattern in schizophrenia onset. J. Neurosci..

[52] Ofori E (2015). Longitudinal changes in free-water within the substantia nigra of Parkinson’s disease. Brain: a J. Neurol..

[53] Zhou G (2023). Monitoring substantia nigra degeneration using free water imaging across prodromal and clinical Parkinson’s disease. Mov. Disord..

[54] Planetta PJ (2016). Free-water imaging in Parkinson’s disease and atypical parkinsonism. Brain: a J. Neurol..

[55] Zhou L (2021). Increased free water in the substantia nigra in idiopathic REM sleep behaviour disorder. Brain: a J. Neurol..

[56] Langkammer C (2012). Quantitative susceptibility mapping (QSM) as a means to measure brain iron? A post mortem validation study. NeuroImage.

[57] Li J (2020). Ferroptosis: past, present and future. Cell Death Dis..

[58] Ostrerova-Golts N (2000). The A53T alpha-synuclein mutation increases iron-dependent aggregation and toxicity. J. Neurosci..

[59] Barbosa JHO (2015). Quantifying brain iron deposition in patients with Parkinson’s disease using quantitative susceptibility mapping, R2 and R2*. Magn. Reson. Imaging.

[60] Langkammer, C. et al. Quantitative susceptibility mapping in Parkinson’s disease. *PLoS ONE* **11**, e0162460 (2016). 10.1371/journal.pone.0162460 PMC5012676 27598250

[61] Acosta-Cabronero J (2017). The whole-brain pattern of magnetic susceptibility perturbations in Parkinson’s disease. Brain: a J. Neurol..

[62] Thomas GEC (2020). Brain iron deposition is linked with cognitive severity in Parkinson’s disease. J. Neurol. Neurosurg. Psychiatry.

[63] Murakami Y (2015). Usefulness of Quantitative Susceptibility Mapping for the Diagnosis of Parkinson Disease. AJNR Am. J. Neuroradiol..

[64] Azuma M (2016). Lateral Asymmetry and Spatial Difference of Iron Deposition in the Substantia Nigra of Patients with Parkinson Disease Measured with Quantitative Susceptibility Mapping. AJNR Am. J. Neuroradiol..

[65] Kim EY (2018). Diagnosis of Early-Stage Idiopathic Parkinson’s Disease Using High-Resolution Quantitative Susceptibility Mapping Combined with Histogram Analysis in the Substantia Nigra at 3 T. J. Clin. Neurol. Seoul. Korea.

[66] Xiao B (2019). Quantitative susceptibility mapping based hybrid feature extraction for diagnosis of Parkinson’s disease. NeuroImage Clin..

[67] Shahmaei V, Faeghi F, Mohammdbeigi A, Hashemi H, Ashrafi F (2019). Evaluation of iron deposition in brain basal ganglia of patients with Parkinson’s disease using quantitative susceptibility mapping. Eur. J. Radiol. Open.

[68] Li G (2019). 3D texture analyses within the substantia nigra of Parkinson’s disease patients on quantitative susceptibility maps and R2∗ maps. NeuroImage.

[69] Sun J (2020). Quantitative Evaluation of Iron Content in Idiopathic Rapid Eye Movement Sleep Behavior Disorder. Mov. Disord..

[70] He N (2021). Imaging iron and neuromelanin simultaneously using a single 3D gradient echo magnetization transfer sequence: Combining neuromelanin, iron and the nigrosome-1 sign as complementary imaging biomarkers in early stage Parkinson’s disease. NeuroImage.

[71] Xiao B (2021). Stability of AI-Enabled Diagnosis of Parkinson’s Disease: A Study Targeting Substantia Nigra in Quantitative Susceptibility Mapping Imaging. Front. Neurosci..

[72] Zhang D (2022). Quantitative susceptibility mapping and free water imaging of substantia nigra in parkinson’s disease. J. Parkinson’s Dis..

[73] Zhang Y (2022). Histogram Analysis of Quantitative Susceptibility Mapping for the Diagnosis of Parkinson’s Disease. Acad. Radiol..

[74] Lancione M (2022). Evaluation of iron overload in nigrosome 1 via quantitative susceptibility mapping as a progression biomarker in prodromal stages of synucleinopathies. Neuroimage.

[75] Rong Y (2022). Combination of Quantitative Susceptibility Mapping and Diffusion Kurtosis Imaging Provides Potential Biomarkers for Early-Stage Parkinson’s Disease. ACS Chem. Neurosci..

[76] Shukla D (2023). Glutathione Depletion and Concomitant Elevation of Susceptibility in Patients with Parkinson’s Disease: State-of-the-Art MR Spectroscopy and Neuropsychological Study. ACS Chem. Neurosci..

[77] Alushaj E (2024). Increased iron in the substantia nigra pars compacta identifies patients with early Parkinson’sdisease: A 3T and 7T MRI study. NeuroImage Clin..

[78] Homayoon N (2019). Nigral iron deposition in common tremor disorders. Mov. Disord..

[79] Cao Q (2023). Diagnostic value of combined magnetic resonance imaging techniques in the evaluation of Parkinson disease. Quant. Imaging Med. Surg..

[80] Pyatigorskaya N (2020). Iron Imaging as a Diagnostic Tool for Parkinson’s Disease: A Systematic Review and Meta-Analysis. Front. Neurol..

[81] Wang Y (2023). An automatic interpretable deep learning pipeline for accurate Parkinson’s disease diagnosis using quantitative susceptibility mapping and T1-weighted images. Hum. Brain Mapp..

[82] Hartono S (2023). Quantitative iron-neuromelanin MRI associates with motor severity in Parkinson’s disease and matches radiological disease classification. Front. Aging Neurosci..

[83] Shin DH (2021). Automated assessment of the substantia nigra on susceptibility map-weighted imaging using deep convolutional neural networks for diagnosis of Idiopathic Parkinson’s disease. Parkinsonism Relat. Disord..

[84] Nepozitek J (2023). Magnetic susceptibility changes in the brainstem reflect REM sleep without atonia severity in isolated REM sleep behavior disorder. NPJ Parkinson’s Dis..

[85] Zecca L (2002). The absolute concentration of nigral neuromelanin, assayed by a new sensitive method, increases throughout the life and is dramatically decreased in Parkinson’s disease. Febs Lett..

[86] Li W (2014). Differential developmental trajectories of magnetic susceptibility in human brain gray and white matter over the lifespan. Hum. Brain Mapp..

[87] Mollenhauer B (2014). Quantification of α-synuclein in cerebrospinal fluid: How ideal is this biomarker for Parkinson’s disease?. Parkinsonism Relat. Disord..

[88] Fairfoul G (2016). Alpha-synuclein RT-QuIC in the CSF of patients with alpha-synucleinopathies. Ann. Clin. Transl. Neurol..

[89] Shahnawaz M (2017). Development of a Biochemical Diagnosis of Parkinson Disease by Detection of α-Synuclein Misfolded Aggregates in Cerebrospinal Fluid. JAMA Neurol..

[90] Concha‐Marambio L (2021). Seed Amplification Assay to Diagnose Early Parkinson’s and Predict Dopaminergic Deficit Progression. Mov. Disord..

[91] Groveman BR (2018). Rapid and ultra-sensitive quantitation of disease-associated α-synuclein seeds in brain and cerebrospinal fluid by αSyn RT-QuIC. Acta Neuropathol. Commun..

[92] Kang UJ (2019). Comparative study of cerebrospinal fluid α-synuclein seeding aggregation assays for diagnosis of Parkinson’s disease. Mov. Disord..

[93] Bargar C (2021). Streamlined alpha-synuclein RT-QuIC assay for various biospecimens in Parkinson’s disease and dementia with Lewy bodies. Acta Neuropathol. Commun..

[94] Rossi M (2020). Ultrasensitive RT-QuIC assay with high sensitivity and specificity for Lewy body-associated synucleinopathies. Acta Neuropathol. (Berl.).

[95] Iranzo A (2021). Detection of α-synuclein in CSF by RT-QuIC in patients with isolated rapid-eye-movement sleep behaviour disorder: a longitudinal observational study. Lancet Neurol..

[96] Bräuer S (2023). Kinetic parameters of alpha-synuclein seed amplification assay correlate with cognitive impairment in patients with Lewy body disorders. Acta Neuropathol. Commun..

[97] Russo MJ (2021). Correction to: High diagnostic performance of independent alpha-synuclein seed amplification assays for detection of early Parkinson’s disease. Acta Neuropathol. Commun..

[98] Siderowf A (2023). Assessment of heterogeneity among participants in the Parkinson’s Progression Markers Initiative cohort using α-synuclein seed amplification: a cross-sectional study. Lancet Neurol..

[99] Brockmann K (2021). Association between CSF alpha-synuclein seeding activity and genetic status in Parkinson’s disease and dementia with Lewy bodies. Acta Neuropathol. Commun..

[100] Garrido A (2019). α-synuclein RT-QuIC in cerebrospinal fluid of LRRK2-linked Parkinson’s disease. Ann. Clin. Transl. Neurol..

[101] Manne S (2020). Blinded RT-QuIC Analysis of α-Synuclein Biomarker in Skin Tissue From Parkinson’s Disease Patients. Mov. Disord. J. Mov. Disord. Soc..

[102] De Luca CMG (2019). Efficient RT-QuIC seeding activity for α-synuclein in olfactory mucosa samples of patients with Parkinson’s disease and multiple system atrophy. Transl. Neurodegener..

[103] Luan M (2022). Diagnostic Value of Salivary Real-Time Quaking-Induced Conversion in Parkinson’s Disease and Multiple System Atrophy. Mov. Disord. J. Mov. Disord. Soc..

[104] Chahine LM (2020). In vivo distribution of α-synuclein in multiple tissues and biofluids in Parkinson disease. Neurology.

[105] Okuzumi A (2023). Propagative α-synuclein seeds as serum biomarkers for synucleinopathies. Nat. Med..

[106] Jiang C (2020). Serum neuronal exosomes predict and differentiate Parkinson’s disease from atypical parkinsonism. J. Neurol. Neurosurg. Psychiatry.

[107] Xylaki M (2023). Extracellular Vesicles for the Diagnosis of Parkinson’s Disease: Systematic Review and Meta-Analysis. Mov. Disord. J. Mov. Disord. Soc..

[108] Stuendl A (2021). α-Synuclein in Plasma-Derived Extracellular Vesicles Is a Potential Biomarker of Parkinson’s Disease. Mov. Disord. J. Mov. Disord. Soc..

[109] Kluge A (2022). Detection of neuron-derived pathological α-synuclein in blood. Brain J. Neurol..

[110] Santaella A (2020). Inflammation biomarker discovery in Parkinson’s disease and atypical parkinsonisms. BMC Neurol..

[111] Bartl M (2023). Blood Markers of Inflammation, Neurodegeneration, and Cardiovascular Risk in Early Parkinson’s Disease. Mov. Disord. J. Mov. Disord. Soc..

[112] Gold L (2010). Aptamer-Based Multiplexed Proteomic Technology for Biomarker Discovery. PLOS ONE.

[113] Posavi M (2019). Characterization of Parkinson’s disease using blood-based biomarkers: A multicohort proteomic analysis. PLoS Med..

[114] Winchester L (2023). Identification of a possible proteomic biomarker in Parkinson’s disease: discovery and replication in blood, brain and cerebrospinal fluid. Brain Commun..

[115] Abdi IY (2023). Cross-sectional proteomic expression in Parkinson’s disease-related proteins in drug-naïve patients vs healthy controls with longitudinal clinical follow-up. Neurobiol. Dis..

[116] Poggiolini I (2022). Diagnostic value of cerebrospinal fluid alpha-synuclein seed quantification in synucleinopathies. Brain.

[117] Kivisäkk P (2024). Clinical evaluation of a novel plasma pTau217 electrochemiluminescence immunoassay in Alzheimer’s disease. Sci. Rep..

[118] Jack CR (2018). NIA-AA Research Framework: Toward a biological definition of Alzheimer’s disease. Alzheimers Dement. J. Alzheimers Assoc..

[119] Laansma MA (2021). International Multicenter Analysis of Brain Structure Across Clinical Stages of Parkinson’s Disease. Mov. Disord..

[120] Kerestes R (2023). Cerebellar Volume and Disease Staging in Parkinson’s Disease: An ENIGMA-PD Study. Mov. Disord..

[121] Lewis MM (2016). The pattern of gray matter atrophy in Parkinson’s disease differs in cortical and subcortical regions. J. Neurol..

[122] Filippi M (2020). Progressive brain atrophy and clinical evolution in Parkinson’s disease. NeuroImage Clin..

[123] Mak E (2015). Baseline and longitudinal grey matter changes in newly diagnosed Parkinson’s disease: ICICLE-PD study. Brain.

[124] Marques, A. et al. Volumetric changes and clinical trajectories in Parkinson’s disease: a prospective multicentric study. *J. Neurol*. 10.1007/s00415-023-11947-0 (2023). 10.1007/s00415-023-11947-0 37648911

[125] Verdi S (2023). Revealing Individual Neuroanatomical Heterogeneity in Alzheimer Disease Using Neuroanatomical Normative Modeling. Neurology.

[126] Bhome, R. et al. A neuroimaging measure to capture heterogeneous patterns of atrophy in Parkinson's disease and dementia with Lewy bodies. *Neuroimage Clin.* **42**, 103596 (2024). 10.1016/j.nicl.2024.103596 PMC10995913 38554485

[127] Abbasi, N. et al. Predicting severity and prognosis in Parkinson’s disease from brain microstructure and connectivity. *NeuroImage Clin*. **25**, 102111 (2020). 10.1016/j.nicl.2019.102111 PMC6926369 31855654

[128] Wang L (2023). Association of Cortical and Subcortical Microstructure With Clinical Progression and Fluid Biomarkers in Patients With Parkinson Disease. Neurology.

[129] Volpicelli-Daley LA (2011). Exogenous α-Synuclein Fibrils Induce Lewy Body Pathology Leading to Synaptic Dysfunction and Neuron Death. Neuron.

[130] Park C (2022). Simulating the progression of brain structural alterations in Parkinson’s disease. Npj Park. Dis..

[131] Pietracupa S (2023). White and gray matter alterations in de novo PD patients: which matter most?. J. Neurol..

[132] Duncan GW (2016). Gray and white matter imaging: A biomarker for cognitive impairment in early Parkinson’s disease?. Mov. Disord..

[133] Kamagata, K. et al. Gray Matter Abnormalities in Idiopathic Parkinson’s Disease: Evaluation by Diffusional Kurtosis Imaging and Neurite Orientation Dispersion and Density Imaging. *Hum. Brain Mapp*. 10.1002/hbm.23628 (2017). 10.1002/hbm.23628 PMC6867088 28470878

[134] Agosta F (2014). Mild cognitive impairment in Parkinson’s disease is associated with a distributed pattern of brain white matter damage. Hum. Brain Mapp..

[135] Minett T (2018). Longitudinal diffusion tensor imaging changes in early Parkinson’s disease: ICICLE-PD study. J. Neurol..

[136] Caspell-Garcia C (2017). Multiple modality biomarker prediction of cognitive impairment in prospectively followed de novo Parkinson disease. PLOS ONE.

[137] Scamarcia PG (2022). Longitudinal White Matter Damage Evolution in Parkinson’s Disease. Mov. Disord..

[138] Tournier J-D, Mori S, Leemans A (2011). Diffusion tensor imaging and beyond. Magn. Reson. Med..

[139] Rau Y-A (2019). A longitudinal fixel-based analysis of white matter alterations in patients with Parkinson’s disease. NeuroImage Clin..

[140] Zarkali, A., McColgan, P., Leyland, L.-A., Lees, A. J. & Weil, R. S. Visual Dysfunction Predicts Cognitive Impairment and White Matter Degeneration in Parkinson’s Disease. *Mov. Disord*. *Mov Disord.* **36**, 1191–1202 (2021). 10.1002/mds.28477 PMC8248368 33421201

[141] Zarkali, A. et al. Neuroimaging and plasma evidence of early white matter loss in Parkinson's disease with poor outcomes. *Brain Commun.* **6**, fcae130 (2024). 10.1093/braincomms/fcae130 PMC11073930 38715714

[142] Mishra VR (2020). Unique white matter structural connectivity in early-stage drug-naive Parkinson disease. Neurology.

[143] Kim YJ (2022). Identifying the white matter structural network of motor reserve in early Parkinson’s disease. Parkinsonism Relat. Disord..

[144] Chung SJ (2022). Association Between White Matter Connectivity and Early Dementia in Patients With Parkinson Disease. Neurology.

[145] Zarkali A (2020). Dementia risk in Parkinson’s disease is associated with interhemispheric connectivity loss and determined by regional gene expression. NeuroImage Clin..

[146] Tessitore A, Cirillo M, De Micco R (2019). Functional Connectivity Signatures of Parkinson’s Disease. J. Park. Dis..

[147] Herz DM, Meder D, Camilleri JA, Eickhoff SB, Siebner HR (2021). Brain Motor Network Changes in Parkinson’s Disease: Evidence from Meta-Analytic Modeling. Mov. Disord. J. Mov. Disord. Soc..

[148] Oldehinkel M (2022). Mapping dopaminergic projections in the human brain with resting-state fMRI. eLife.

[149] Baggio HC, Segura B, Junque C (2015). Resting-state functional brain networks in Parkinson’s disease. CNS Neurosci. Ther..

[150] Fiorenzato E (2019). Dynamic functional connectivity changes associated with dementia in Parkinson’s disease. Brain.

[151] Díez-Cirarda M (2018). Dynamic functional connectivity in Parkinson’s disease patients with mild cognitive impairment and normal cognition. NeuroImage Clin..

[152] Zarkali A (2021). Organisational and neuromodulatory underpinnings of structural-functional connectivity decoupling in patients with Parkinson’s disease. Commun. Biol..

[153] Rabini G (2023). Connectome-based fingerprint of motor impairment is stable along the course of Parkinson’s disease. Cereb. Cortex N. Y. N. 1991.

[154] Cabeza R (2018). Maintenance, reserve and compensation: the cognitive neuroscience of healthy ageing. Nat. Rev. Neurosci..

[155] Johansson, M. E., Toni, I., Kessels, R. P. C., Bloem, B. R. & Helmich, R. C. Clinical severity in Parkinson’s disease is determined by decline in cortical compensation. *Brain* awad325 10.1093/brain/awad325 (2023). 10.1093/brain/awad325 PMC10907095 37757883

[156] Hijazi Z, Yassi N, O’Brien JT, Watson R (2022). The influence of cerebrovascular disease in dementia with Lewy bodies and Parkinson’s disease dementia. Eur. J. Neurol..

[157] Zhao W (2023). Effects of white matter hyperintensity on cognitive function in PD patients: a meta-analysis. Front. Neurol..

[158] Carvalho de Abreu DC (2023). White matter hyperintensity burden predicts cognitive but not motor decline in Parkinson’s disease: results from the Ontario Neurodegenerative Diseases Research Initiative. Eur. J. Neurol..

[159] Dadar M (2018). White matter hyperintensities are linked to future cognitive decline in de novo Parkinson’s disease patients. NeuroImage Clin..

[160] Wan H, Chen H, Zhang M, Feng T, Wang Y (2023). Cerebral microbleeds is associated with dementia in Parkinson’s disease. Acta Neurol. Belg..

[161] Chen K (2022). Lacunes may worsen cognition but not motor function in Parkinson’s disease. Brain Behav..

[162] Chiaravalloti A (2012). Different patterns of cardiac sympathetic denervation in tremor-type compared to akinetic-rigid-type Parkinson’s disease: Molecular imaging with 123I-MIBG. Mol. Med. Rep..

[163] Matsui H (2006). Impaired Visual Acuity as a Risk Factor for Visual Hallucinations in Parkinson’s Disease. J. Geriatr. Psychiatry Neurol..

[164] Rascol O, Schelosky L (2009). 123I-metaiodobenzylguanidine scintigraphy in Parkinson’s disease and related disorders. Mov. Disord..

[165] Park DG, Kang J, An Y-S, Chang J, Yoon JH (2022). Association of plasma α-synuclein with cardiac 123I-MIBG scintigraphy in early Parkinson’s disease. Neurosci. Lett..

[166] Giannoccaro MP (2020). Comparison of 123I-MIBG scintigraphy and phosphorylated α-synuclein skin deposits in synucleinopathies. Parkinsonism Relat. Disord..

[167] Totsune, T. et al. Nuclear Imaging Data-Driven Classification of Parkinson’s Disease. *Mov. Disord. Off. J. Mov. Disord. Soc*. 10.1002/mds.29582 (2023). 10.1002/mds.29582 37638533

[168] Liu Q (2023). An investigation of neuromelanin distribution in substantia nigra and locus coeruleus in patients with Parkinson’s disease using neuromelanin-sensitive MRI. BMC Neurol..

[169] Matsuura K (2016). A longitudinal study of neuromelanin-sensitive magnetic resonance imaging in Parkinson’s disease. Neurosci. Lett..

[170] Ravanfar P (2021). Systematic review: Quantitative susceptibility mapping (QSM) of brain iron profile in neurodegenerative diseases. Front. Neurosci..

[171] Uchida Y (2020). Magnetic susceptibility associates with dopaminergic deficits and cognition in Parkinson’s disease. Mov. Disord..

[172] Thomas, G. E. C., Hannaway, N., Zarkali, A., Shmueli, K. & Weil, R. S. Longitudinal Associations of Magnetic Susceptibility with Clinical Severity in Parkinson’s Disease. *Mov. Disord* **39**, 546–559 (2020). 10.1002/mds.29702 PMC11141787 38173297

[173] Bergsland N (2019). Ventral posterior substantia nigra iron increases over 3 years in Parkinson’s disease. Mov. Disord..

[174] Du G (2018). Distinct progression pattern of susceptibility MRI in the substantia nigra of Parkinson’s patients. Mov. Disord.: Off. J. Mov. Disord. Soc..

[175] Guan X (2020). Asymmetrical nigral iron accumulation in Parkinson’s disease with motor asymmetry: An explorative, longitudinal and test-retest study. *Stress and*. Aging Brain.

[176] Ulla M (2013). Is r2* a new MRI biomarker for the progression of Parkinson’s disease? A longitudinal follow-up. PLoS ONE.

[177] Wieler M, Gee M, Martin WRW (2015). Longitudinal midbrain changes in early Parkinson’s disease: Iron content estimated from R2*/MRI. Parkinsonism Relat. Disord..

[178] Burciu RG (2017). Progression marker of Parkinson’s disease: a 4-year multi-site imaging study. Brain J. Neurol..

[179] Schenck JF (1996). The role of magnetic susceptibility in magnetic resonance imaging: MRI magnetic compatibility of the first and second kinds. Med. Phys..

[180] Zhao Z (2021). The effect of beta-amyloid and tau protein aggregations on magnetic susceptibility of anterior hippocampal laminae in Alzheimer’s diseases. NeuroImage.

[181] Irwin DJ (2020). Evolution of Alzheimer’s Disease Cerebrospinal Fluid Biomarkers in Early Parkinson’s Disease. Ann. Neurol..

[182] Schrag A, Siddiqui UF, Anastasiou Z, Weintraub D, Schott JM (2017). Clinical variables and biomarkers in prediction of cognitive impairment in patients with newly diagnosed Parkinson’s disease: a cohort study. Lancet Neurol..

[183] Batzu L (2022). Plasma p-tau181, neurofilament light chain and association with cognition in Parkinson’s disease. Npj Park. Dis..

[184] Palmqvist S (2020). Discriminative Accuracy of Plasma Phospho-tau217 for Alzheimer Disease vs Other Neurodegenerative Disorders. JAMA.

[185] Lerche S (2020). CSF NFL in a Longitudinally Assessed PD Cohort: Age Effects and Cognitive Trajectories. Mov. Disord. J. Mov. Disord. Soc..

[186] Aamodt WW (2021). Neurofilament Light Chain as a Biomarker for Cognitive Decline in Parkinson Disease. Mov. Disord. J. Mov. Disord. Soc..

[187] Zetterberg H (2016). Association of Cerebrospinal Fluid Neurofilament Light Concentration With Alzheimer Disease Progression. JAMA Neurol..

[188] Sjögren M (2001). Neurofilament protein in cerebrospinal fluid: a marker of white matter changes. J. Neurosci. Res..

[189] Hansson O (2017). Blood-based NfL: A biomarker for differential diagnosis of parkinsonian disorder. Neurology.

[190] Youssef P (2023). Evaluation of plasma levels of NFL, GFAP, UCHL1 and tau as Parkinson’s disease biomarkers using multiplexed single molecule counting. Sci. Rep..

[191] Huh YE (2021). Glucosylceramide in cerebrospinal fluid of patients with GBA-associated and idiopathic Parkinson’s disease enrolled in PPMI. Npj Park. Dis..

[192] Parnetti L (2017). Cerebrospinal fluid β-glucocerebrosidase activity is reduced in Parkinson’s disease patients. Mov. Disord..

[193] Qu Y (2023). A systematic review and meta-analysis of inflammatory biomarkers in Parkinson’s disease. Npj Park. Dis..

[194] Latourelle JC (2017). Large-scale identification of clinical and genetic predictors of motor progression in patients with newly diagnosed Parkinson’s disease: a longitudinal cohort study and validation. Lancet Neurol..

[195] Mollenhauer B (2019). Baseline predictors for progression 4 years after Parkinson’s disease diagnosis in the De Novo Parkinson Cohort (DeNoPa). Mov. Disord. J. Mov. Disord. Soc..

[196] Chen J (2023). Predictors of cognitive impairment in newly diagnosed Parkinson’s disease with normal cognition at baseline: A 5-year cohort study. Front. Aging Neurosci..

[197] Weil, R. S. *et al*. Visual dysfunction in Parkinson’s disease. *Brain J. Neurol*. 10.1093/brain/aww175 (2016). 10.1093/brain/aww175 PMC5091042 27412389

[198] Hamedani, A. G., Abraham, D. S., Maguire, M. G. & Willis, A. W. Visual Impairment Is More Common in Parkinson’s Disease and Is a Risk Factor for Poor Health Outcomes. *Mov. Disord. Off. J. Mov. Disord. Soc*. 10.1002/mds.28182 (2020). 10.1002/mds.28182 PMC8183672 32662528

[199] Lee J-Y (2022). Multimodal brain and retinal imaging of dopaminergic degeneration in Parkinson disease. Nat. Rev. Neurol..

[200] Chrysou A, Jansonius NM, van Laar T (2019). Retinal layers in Parkinson’s disease: A meta-analysis of spectral-domain optical coherence tomography studies. Parkinsonism Relat. Disord..

[201] Murueta-Goyena A (2021). Retinal Thickness Predicts the Risk of Cognitive Decline in Parkinson Disease. Ann. Neurol..

[202] Hannaway, N. et al. Visual dysfunction is a better predictor than retinal thickness for dementia in Parkinson’s disease. *J. Neurol. Neurosurg. Psych.*10.1136/jnnp-2023-331083 (2023). 10.1136/jnnp-2023-331083 PMC10447370 37080759

[203] Wagner SK (2023). Retinal Optical Coherence Tomography Features Associated With Incident and Prevalent Parkinson Disease. Neurology.

[204] Oxtoby NP (2021). Sequence of clinical and neurodegeneration events in Parkinson’s disease progression. Brain J. Neurol..

[205] Parnetti L (2019). CSF and blood biomarkers for Parkinson’s disease. Lancet Neurol..

[206] Majbour NK (2016). Longitudinal changes in CSF alpha-synuclein species reflect Parkinson’s disease progression. Mov. Disord..

[207] Williams SM, Schulz P, Sierks MR (2016). Oligomeric α-synuclein and β-amyloid variants as potential biomarkers for Parkinson’s and Alzheimer’s diseases. Eur. J. Neurosci..

[208] Korat S (2021). Alpha-synuclein PET tracer development-an overview about current efforts. Pharm. (Basel, Switz.).

[209] Xiang J (2023). Development of an alpha-synuclein positron emission tomography tracer for imaging synucleinopathies. Cell.

[210] Smith R (2023). The alpha-synuclein PET tracer [18F] ACI-12589 distinguishes multiple system atrophy from other neurodegenerative diseases. Nat. Commun..

[211] Edison P (2008). Amyloid load in Parkinson’s disease dementia and Lewy body dementia measured with [11C]PIB positron emission tomography. J. Neurol. Neurosurg. Psych..

[212] Johansson A (2008). [(11)C]-PIB imaging in patients with Parkinson’s disease: preliminary results. Parkinsonism Relat. Disord..

[213] Frey KA, Petrou M (2015). Imaging amyloidopathy in Parkinson disease and Parkinsonian dementia syndromes. Clin. Transl. imaging.

[214] Baik K (2023). Effect of amyloid on cognitive performance in Parkinson’s disease and dementia with lewy bodies. Mov. Disord.: Off. J. Mov. Disord. Soc..

[215] Ghadery C (2020). The interaction between neuroinflammation and beta-amyloid in cognitive decline in Parkinson’s disease. Mol. Neurobiol..

[216] Mihaescu AS (2022). Beta amyloid deposition and cognitive decline in Parkinson’s disease: a study of the PPMI cohort. Mol. Brain.

[217] Gomperts SN (2013). Amyloid is linked to cognitive decline in patients with Parkinson disease without dementia. Neurology.

[218] Zhang J, Jin J, Su D, Feng T, Zhao H (2023). Tau-PET imaging in Parkinson’s disease: a systematic review and meta-analysis. Front. Neurol..

[219] Mueller A (2020). Tau PET imaging with (18)F-PI-2620 in patients with Alzheimer disease and healthy controls: A first-in-humans study. J. Nucl. Med.: Off. Publ., Soc. Nucl. Med..

[220] Gomperts SN (2016). Tau positron emission tomographic imaging in the lewy body diseases. JAMA Neurol..

[221] Lee SH (2018). Distinct patterns of amyloid-dependent tau accumulation in Lewy body diseases. Mov. Disord.: Off. J. Mov. Disord. Soc..

[222] Buongiorno M (2017). Cross-Sectional and Longitudinal Cognitive Correlates of FDDNP PET and CSF Amyloid-β and Tau in Parkinson’s Disease1. J. Alzheimers Dis. JAD.

[223] Lobanova E (2021). Imaging protein aggregates in the serum and cerebrospinal fluid in Parkinson’s disease. Brain.

[224] Bachhuber T (2015). Inhibition of amyloid-β plaque formation by α-synuclein. Nat. Med..

[225] Guo JL (2013). Distinct alpha-synuclein strains differentially promote tau inclusions in neurons. Cell.

[226] Grober E (1999). Memory and mental status correlates of modified Braak staging. Neurobiol. Aging.

[227] Mashima K (2017). Extremely low prevalence of amyloid positron emission tomography positivity in Parkinson’s disease without dementia. Eur. Neurol..

[228] De Miranda, B. R., Van Houten, B. & Sanders, L. H. Toxin-Mediated Complex I Inhibition and Parkinson’s Disease. in *Mitochondrial Mechanisms of Degeneration and Repair in Parkinson’s Disease* (ed. Buhlman, L. M.) 115–137 (Springer International Publishing, Cham). 10.1007/978-3-319-42139-1_6 (2016).

[229] Sanders LH (2017). Editor’s Highlight: Base Excision Repair Variants and Pesticide Exposure Increase Parkinson’s Disease Risk. Toxicol. Sci..

[230] Sanders LH (2014). Mitochondrial DNA damage: Molecular marker of vulnerable nigral neurons in Parkinson’s disease. Neurobiol. Dis..

[231] von Linstow CU, Gan-Or Z, Brundin P (2020). Precision medicine in Parkinson’s disease patients with LRRK2 and GBA risk variants - Let’s get even more personal. Transl. Neurodegener..

[232] Qi R (2023). A blood-based marker of mitochondrial DNA damage in Parkinson’s disease. Sci. Transl. Med..

[233] Roussakis AA, Piccini P (2018). Molecular imaging of neuroinflammation in idiopathic Parkinson’s disease. Int. Rev. Neurobiol..

[234] Edison P (2013). Microglia, amyloid, and glucose metabolism in Parkinson’s disease with and without dementia. Neuropsychopharmacol.: Off. Publ. Am. Coll. Neuropsychopharmacol..

[235] Iannaccone S (2013). In vivo microglia activation in very early dementia with Lewy bodies, comparison with Parkinson’s disease. Parkinsonism Relat. Disord..

[236] Nicastro N, Surendranathan A, Mak E, Rowe JB, O’Brien JT (2019). (11) C-PK11195 PET imaging and white matter changes in Parkinson’s disease dementia. Ann. Clin. Transl. Neurol..

[237] Bae K-R, Shim H-J, Balu D, Kim SR, Yu S-W (2014). Translocator protein 18 kDa negatively regulates inflammation in microglia. J. NeuroImmune Pharmacol. J. Soc. NeuroImmune Pharmacol..

[238] Nutma E (2023). Translocator protein is a marker of activated microglia in rodent models but not human neurodegenerative diseases. Nat. Commun..

[239] Tan, J. B. et al. Abnormal higher-order network interactions in Parkinson’s disease visual hallucinations. *Brain* awad305 10.1093/brain/awad305 (2023). 10.1093/brain/awad305 37677056

[240] Stiso J (2019). White Matter Network Architecture Guides Direct Electrical Stimulation through Optimal State Transitions. Cell Rep..

[241] Medaglia JD (2021). Language Tasks and the Network Control Role of the Left Inferior Frontal Gyrus. eNeuro.

[242] Zarkali A (2020). Differences in network controllability and regional gene expression underlie hallucinations in Parkinson’s disease. Brain.

[243] Neudorfer C (2023). Lead-DBS v3.0: Mapping deep brain stimulation effects to local anatomy and global networks. NeuroImage.

[244] Tian YE (2023). Heterogeneous aging across multiple organ systems and prediction of chronic disease and mortality. Nat. Med..

[245] Romano S (2021). Meta-analysis of the Parkinson’s disease gut microbiome suggests alterations linked to intestinal inflammation. Npj Park. Dis..

[246] Nowak JM, Kopczyński M, Friedman A, Koziorowski D, Figura M (2022). Microbiota Dysbiosis in Parkinson Disease—In Search of a Biomarker. Biomedicines.

[247] Drori E, Berman S, Mezer AA (2022). Mapping microstructural gradients of the human striatum in normal aging and Parkinson’s disease. Sci. Adv..

[248] Filo S (2023). Non-invasive assessment of normal and impaired iron homeostasis in the brain. Nat. Commun..

[249] Lesbats C (2021). High-frequency electrical properties tomography at 9.4T as a novel contrast mechanism for brain tumors. Magn. Reson. Med..

[250] Park S (2022). Application of High-Frequency Conductivity Map Using MRI to Evaluate It in the Brain of Alzheimer’s Disease Patients. Front. Neurol..

[251] Schulz-Schaeffer WJ (2010). The synaptic pathology of α-synuclein aggregation in dementia with Lewy bodies, Parkinson’s disease and Parkinson’s disease dementia. Acta Neuropathol..

[252] Martin, S. L., Uribe, C. & Strafella, A. P. PET imaging of synaptic density in Parkinsonian disorders. *Journal of neuroscience research*10.1002/jnr.25253 (2023). 10.1002/jnr.25253 37814917

[253] Deistung A (2013). High-Resolution MR Imaging of the Human Brainstem In vivo at 7 Tesla. Front. Hum. Neurosci..

[254] Plantinga BR (2018). Individualized parcellation of the subthalamic nucleus in patients with Parkinson’s disease with 7T MRI. NeuroImage.

[255] Kwon D-H (2012). Seven-Tesla magnetic resonance images of the substantia nigra in Parkinson disease. Ann. Neurol..

[256] Poston KL (2020). Substantia Nigra Volume Dissociates Bradykinesia and Rigidity from Tremor in Parkinson’s Disease: A 7 Tesla Imaging Study. J. Park. Dis..

[257] Patriat R (2020). Morphological changes in the subthalamic nucleus of people with mild-to-moderate Parkinson’s disease: a 7T MRI study. Sci. Rep..

[258] Wolters AF (2023). Neuromelanin related ultra-high field signal intensity of the locus coeruleus differs between Parkinson’s disease and controls. NeuroImage Clin..

[259] Ye R (2022). Locus Coeruleus Integrity from 7 T MRI Relates to Apathy and Cognition in Parkinsonian Disorders. Mov. Disord. J. Mov. Disord. Soc..

[260] Mathiopoulou V (2023). Utilizing 7-Tesla Subthalamic Nucleus Connectivity in Deep Brain Stimulation for Parkinson Disease. Neuromodulation J. Int. Neuromodulation Soc..

[261] McColgan, P. et al. Relating quantitative 7T MRI across cortical depths to cytoarchitectonics, gene expression and connectomics: a framework for tracking neurodegenerative disease. *bioRxiv* 2020.02.05.935080 10.1101/2020.02.05.935080 (2020). 10.1002/hbm.25595 PMC8449108 34272784

[262] Pasquereau B, DeLong MR, Turner RS (2016). Primary motor cortex of the Parkinsonian monkey: altered encoding of active movement. Brain J. Neurol..

[263] Boulant N (2023). Commissioning of the Iseult CEA 11.7 T whole-body MRI: current status, gradient–magnet interaction tests and first imaging experience. Magn. Reson. Mater. Phys. Biol. Med..

[264] Bates S (2023). A vision of 14 T MR for fundamental and clinical science. Magn. Reson. Mater. Phys. Biol. Med..

